# Functional profiling of COVID-19 respiratory tract microbiomes

**DOI:** 10.1038/s41598-021-85750-0

**Published:** 2021-03-19

**Authors:** Niina Haiminen, Filippo Utro, Ed Seabolt, Laxmi Parida

**Affiliations:** 1 IBM T. J. Watson Research Center, Yorktown Heights, NY USA; 2 IBM Almaden Research Center, San Jose, CA USA

**Keywords:** Computational biology and bioinformatics, Metagenomics

## Abstract

In response to the ongoing global pandemic, characterizing the molecular-level host interactions of the new coronavirus SARS-CoV-2 responsible for COVID-19 has been at the center of unprecedented scientific focus. However, when the virus enters the body it also interacts with the micro-organisms already inhabiting the host. Understanding the virus-host-microbiome interactions can yield additional insights into the biological processes perturbed by viral invasion. Alterations in the gut microbiome species and metabolites have been noted during respiratory viral infections, possibly impacting the lungs via gut-lung microbiome crosstalk. To better characterize microbial functions in the lower respiratory tract during COVID-19 infection, we carry out a functional analysis of previously published metatranscriptome sequencing data of bronchoalveolar lavage fluid from eight COVID-19 cases, twenty-five community-acquired pneumonia patients, and twenty healthy controls. The functional profiles resulting from comparing the sequences against annotated microbial protein domains clearly separate the cohorts. By examining the associated metabolic pathways, distinguishing functional signatures in COVID-19 respiratory tract microbiomes are identified, including decreased potential for lipid metabolism and glycan biosynthesis and metabolism pathways, and increased potential for carbohydrate metabolism pathways. The results include overlap between previous studies on COVID-19 microbiomes, including decrease in the glycosaminoglycan degradation pathway and increase in carbohydrate metabolism. The results also suggest novel connections to consider, possibly specific to the lower respiratory tract microbiome, calling for further research on microbial functions and host-microbiome interactions during SARS-CoV-2 infection.

## Introduction

An impressive number of scientific studies have rapidly been published on the genomics and molecular-level host interactions of the respiratory coronavirus SARS-CoV-2^1^ of reported bat origin^2^, responsible for the COVID-19 disease pandemic. In addition to characterizing the process of viral infection and host response^3^, understanding changes in the microenvironment within the host can yield further insights into the perturbed biological processes^4^ and their connections with disease risk factors^5^. The gut and lungs are closely linked organs that affect each other via an immunological co-ordination between them, and microbes have a central role in shaping the normal and pathologic immune responses in both^6^. Microbiome-mediated cross-talk along the gut-lung axis has been noted during lung infection specifically due to alterations in the gut microbial species and metabolites^7,8^. The gut microbiota has a critical role in pulmonary immunity and the host’s defense against viral respiratory infections; current evidence points to SARS-CoV-2 infection altering the gut barrier, leading to the systemic spread of bacteria, endotoxins, and microbial metabolites^9^. It has been suggested that a cycle between SARS-CoV2 infection, systemic inflammation, disrupted intestinal barrier integrity, and microbial translocation contributes to COVID-19 severity^10^.

The respiratory microbiome during SARS-CoV-2 infection has also been under investigation^1,11–13^. Previous studies on the respiratory tract microbiome during other pathogen infections have examined its predictivity of clinical outcomes, and associated potential probiotic interventions^14–18^. In a study of the oropharyngeal microbiome, reduced microbiome diversity and high dysbiosis were observed in hospitalized patients with severe COVID-19, associated with a loss of microbial genes and metabolic pathways^19^. It has also been demonstrated that SARS-CoV-2 causes a significant change in the microbiome present in nasopharyngeal specimens^20^. The upper respiratory tract has been investigated for co-infection of other pathogens and SARS-CoV-2^21^, while alterations in its microbiota has been observed in COVID-19 patients and associated with the fatality rate^22^.

To better understand the role of the lower respiratory microbiome in COVID-19, we introduce a *functional* analysis, as opposed to taxonomic naming, from a collection of metatranscriptomes from bronchoalveolar lavage fluid (BALF) of COVID-19 patients, healthy subjects, and community-acquired pneumonia (CAP) cases^11^. While Shen et al.^11^ focused on the SARS-CoV-2 genomes and taxonomic profiling of the microbiomes, here we perform global functional profiling to characterize altered biological processes in the respiratory tract microbiomes. Our protein domain focused amino acid matching approach supports the profiling of microbial functions performed by known and potentially unknown organisms yet to be characterized^23^. The robust comparative analysis presented here was designed to highlight consistent differences in COVID-19 patient microbiomes compared to both community-acquired pneumonia and healthy control samples.

## Results

### Functional profiling framework

**Figure 1:**
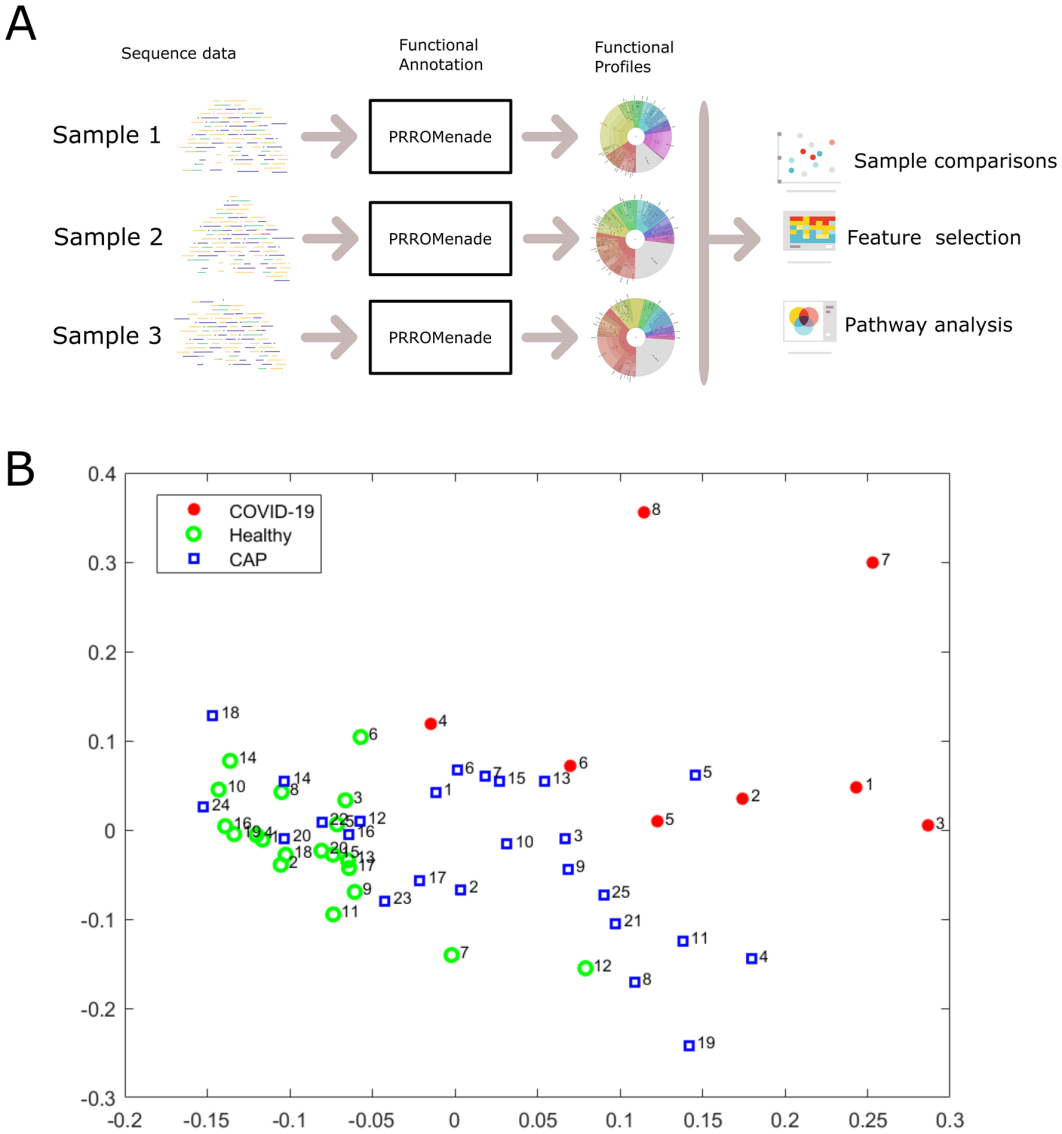
Overall analysis workflow and two-dimensional projection of functional profiles. (**A**). Each microbiome sequencing sample is annotated with PRROMenade, utilizing labeled reference data from the IBM Functional Genomic Platform. The resulting functional profiles are visualized and compared in downstream analyses. (**B**). Multidimensional scaling of the functional profiles using the Spearman distance. Each sample is represented by a marker colored by cohort and labeled by the sample number within that cohort.

The overall analysis workflow is depicted in Fig. 1A. The total RNA sequencing reads were first trimmed and filtered, followed by translation and functional classification with PRROMenade^24^ against a vast amino acid sequence collection of 21 million bacterial and viral protein domains from the IBM Functional Genomics Platform^25^, annotated with KEGG enzyme codes (EC) from a corresponding functional hierarchy^26^ (see Supplementary Fig. S1 for filtering results). PRROMenade has previously been applied in functional annotation of gut^24^ and soil microbiomes^27^. Post-processing and robust rank-based RoDEO^28^ projection onto a unified scale was performed to make the resulting functional profiles comparable.

### Microbiome functional profiles cluster by cohort

While the individual functional profiles vary, a robust comparative analysis reveals specific functions that are consistently altered between cohorts (Supplementary File S1 shows a Krona^29^ visualization of each sample). The read counts assigned at various functional hierarchy levels (Supplementary Fig. S2) were pushed down to the leaf level, and very low abundant features were removed for subsequent analyses (see Methods).

Multi-dimensional scaling of pairwise Spearman distances between the samples separates the COVID-19 cohort, while CAP samples are located between healthy and COVID-19 samples (Fig. 1B). A significant difference between the functional profiles was observed between the COVID-19, CAP, and healthy control cohorts according to the PERMANOVA test ($p \le 0.0001$). Functional profiling separated the cohorts with a similar score as taxonomic profiling (functional profiling $R^2=0.06$ vs. taxonomic profiling by Shen et al.^11^ $R^2 = 0.07$).

### Differentially abundant features distinguish COVID-19 samples

The RNA sequencing data had varying total number of reads and human content per sample (Supplementary Fig. S1). Therefore we used RoDEO^28^ to project the functional profiles onto a robust uniform scale. To examine the most differentiating features for COVID-19 versus the other cohorts, we extracted 30 top-ranked features from the COVID vs. CAP comparison and from the COVID vs. healthy controls comparison. We then considered the union of the feature sets, resulting in 44 EC features.

**Figure 2:**
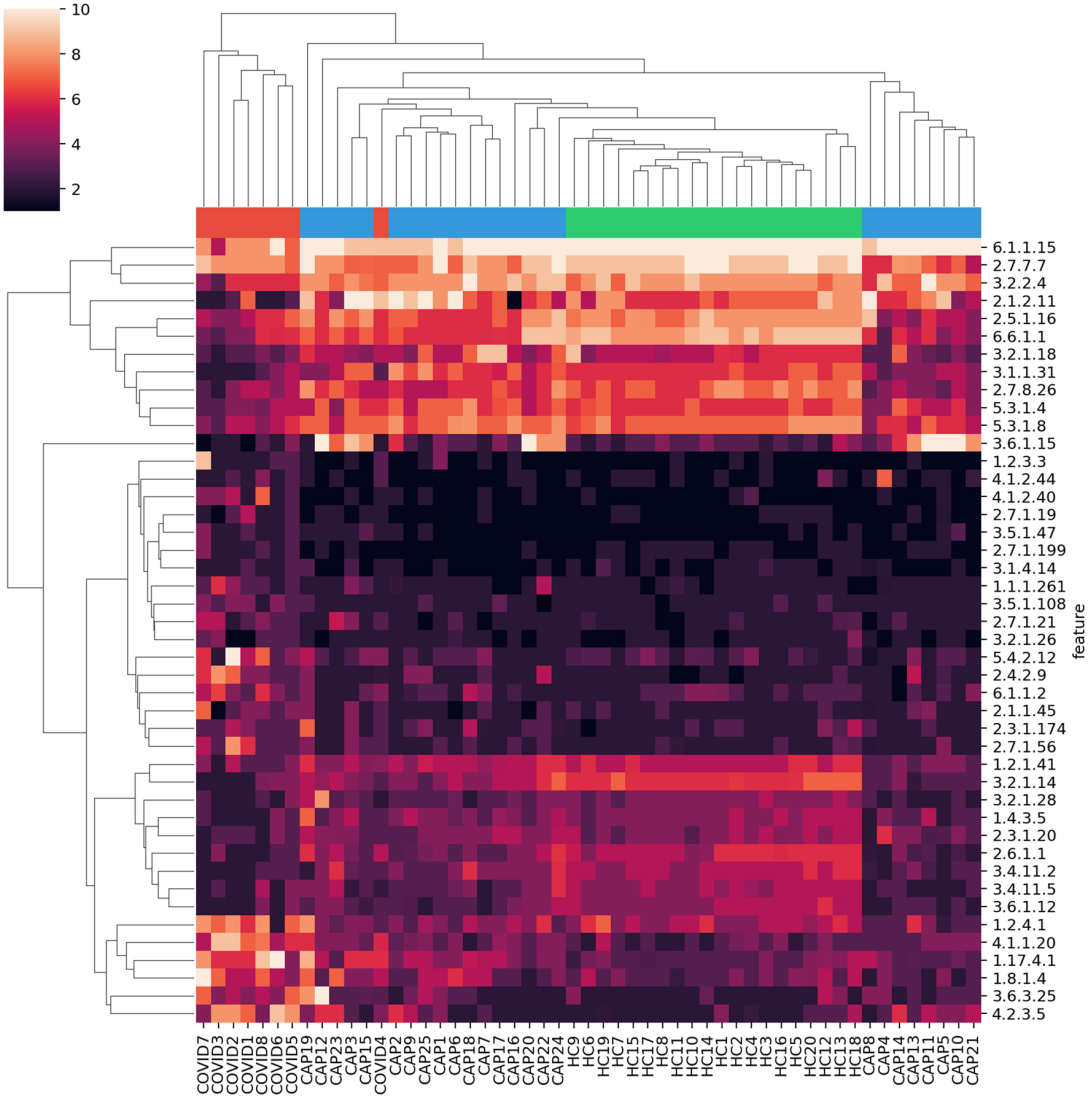
Clustering with top differentiating functional features. RoDEO processed EC abundance values (10 denotes highest possible value), for 44 features differentiating COVID-19 from community-acquired pneumonia and healthy controls. Columns and rows are ordered independently by hierarchical clustering of features and samples. The colors attached to the dendrogram on top reflect the cohort labels: red = COVID-19, blue = CAP, green = healthy control.

When clustering the samples using the top differentiating features, the COVID-19 samples are grouped together and separate from the other cohorts, except for sample 4 (Fig. 2). While the examined 44 features were selected as those differentiating COVID-19 from CAP and healthy controls, they also separate the healthy control samples from all others; the healthy controls cluster tightly together. The results also demonstrate that the samples do not merely cluster by the total number of input reads or the fraction of functionally annotated microbial reads, since those measures vary within cohorts (Supplementary Fig. S1). The CAP patient samples were collected from different hospital sources, prior to the current pandemic, and represent pneumonia cases with various viruses detected in the sequencing data^11^ (e.g. enterovirus, influenza virus, rhinovirus), possibly contributing to the greater variability between their microbiome functional profiles.

The COVID-19 samples have more abundant EC features including (see bottom left feature cluster in Fig. 2) 1.2.4.1 “Pyruvate dehydrogenase”, 4.1.1.20 “Diaminopimelate decarboxylase”, 1.17.4.1 “Ribonucleoside-diphosphate reductase”, 1.8.1.4 “Dihydrolipoyl dehydrogenase”, 3.6.3.25 “Sulfate-transporting ATPase”, and 4.2.3.5 “Chorismate synthase”, linked to various amino acid, carbohydrate, energy, and nucleotide metabolism pathways. EC 4.1.1.20 was also detected as increased in a metaproteome study of COVID-19 respiratory microbiomes^30^. Supplementary Fig. S3 includes a scatter plot of the average change per EC in COVID-19 compared to CAP and healthy cohorts, highlighting outliers.

### Altered lung microbiome pathways indicated in COVID-19

**Figure 3:**
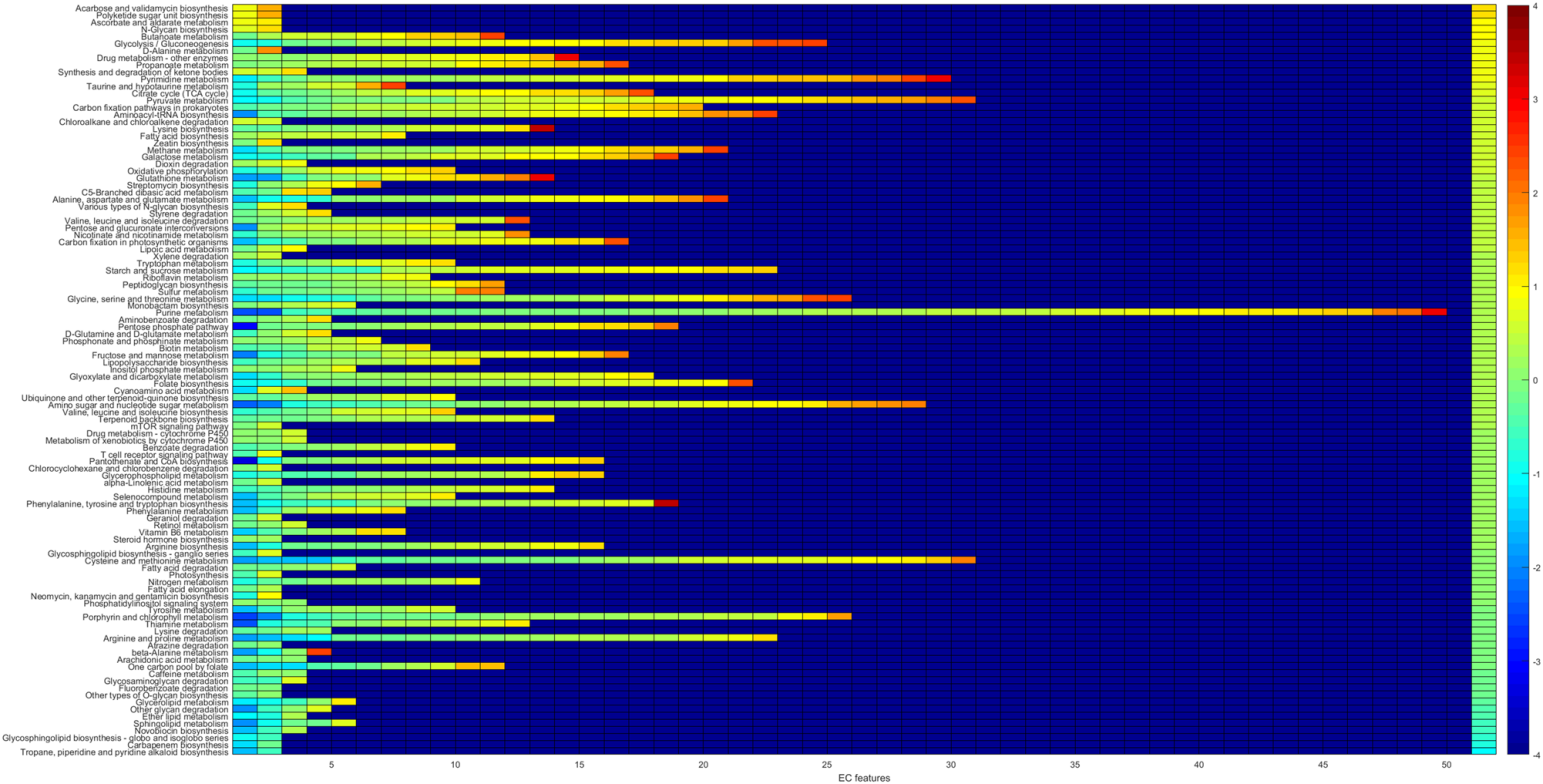
Pathway changes in COVID-19 samples. For each pathway (row), there are as many entries as there are detected EC features. The color of the entries indicate the average of COVID-19 versus CAP and COVID-19 versus HC changes. The entries on each row are ordered from low to high values. The background value (dark blue) indicates no data; some pathways have more detected features than others (only pathways with at least two EC features detected are considered). The rightmost column indicates the pathway score, the pathways are ordered accordingly from top (higher in COVID-19) to bottom (lower in COVID-19).

**Figure 4:**
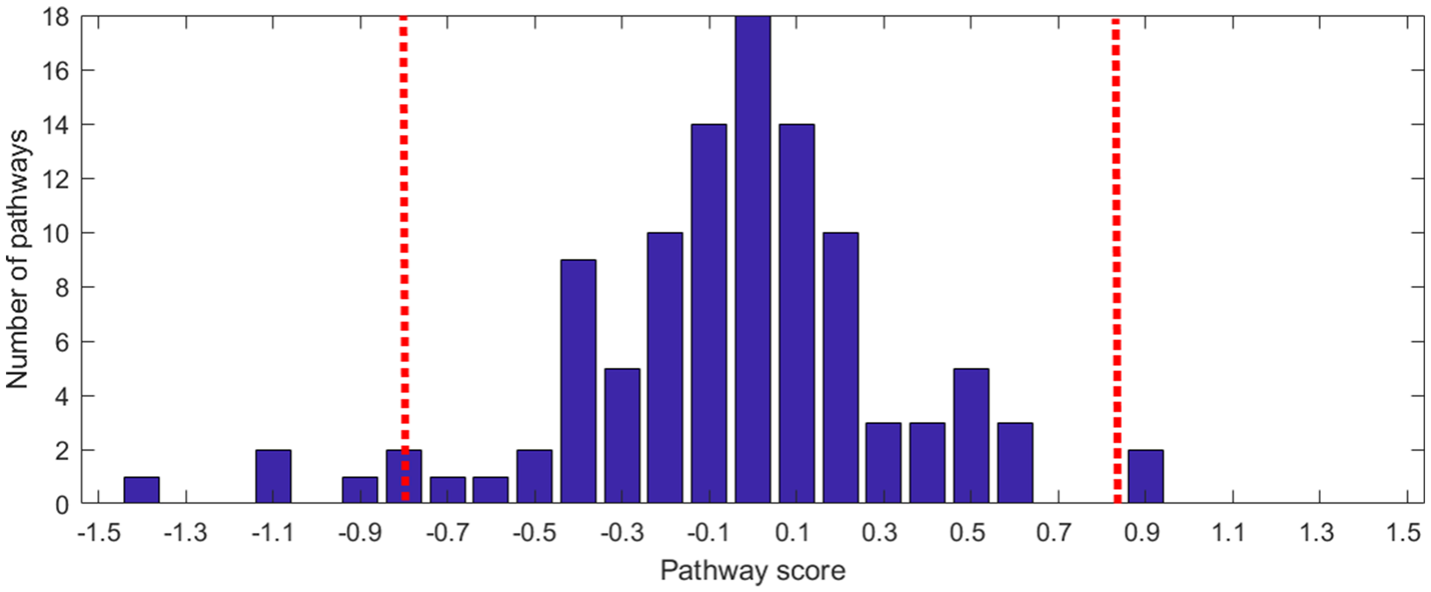
Pathway score distribution. The histogram of observed pathway scores is shown. The score thresholds for determining outliers according to median absolute deviation (MAD) is also marked (red dashed lines, three scaled median deviations away from the median).

In order to systematically examine the detected features against functional pathways, all the EC features were considered against their corresponding pathways from the KEGG metabolic pathway mapping^26^. Pathway scores (mean abundance change in COVID-19) were computed using all the detected EC features per pathway, see Fig. 3. To identify outlying pathway scores (high or low compared to the observed distribution), median absolute deviation (MAD)^31^, a robust measure of dispersion was utilized, see Fig. 4. The most differential pathways are shown in Table 1.

**Table 1:** Altered pathways in COVID-19 The most differential pathways in COVID-19 (with dist. $\ge 2$) are shown in the table.

Pathway name	dist.	+/−	#EC	Type
Tropane, piperidine and pyridine alkaloid biosynthesis	5.04*	−	2	Biosynthesis of other secondary metabol.
Carbapenem biosynthesis	3.84*	−	2	Biosynthesis of other secondary metabol.
Glycosphingolipid biosynth. - globo and isoglobo series	3.75*	−	2	Glycan biosynthesis and metabolism
Novobiocin biosynthesis	3.28*	−	3	Biosynthesis of other secondary metabol.
Sphingolipid metabolism	2.90	−	5	Lipid metabolism
Ether lipid metabolism	2.82	−	3	Lipid metabolism
Other glycan degradation	2.64	−	4	Glycan biosynthesis and metabolism
Glycerolipid metabolism	2.11	−	5	Lipid metabolism
Acarbose and validamycin biosynthesis	3.47*	+	2	Biosynthesis of other secondary metabol.
Polyketide sugar unit biosynthesis	3.47*	+	2	Metabolism of terpenoids and polyketides
Ascorbate and aldarate metabolism	2.45	+	2	Carbohydrate metabolism
N-Glycan biosynthesis	2.38	+	2	Glycan biosynthesis and metabolism
Butanoate metabolism	2.33	+	11	Carbohydrate metabolism
Glycolysis / Gluconeogenesis	2.09	+	24	Carbohydrate metabolism
D-Alanine metabolism	2.04	+	2	Metabolism of other amino acids

Here dist. denotes the pathway score’s distance from the median, divided by the scaled median absolute deviation. Pathways that are determined outliers (dist. $\ge 3$) are marked with *. The direction of the change in COVID-19 (+/−), the number of associated EC features per pathway detected from functional profiling (#EC), and the pathway type are also shown.

Among the pathways lower in COVID-19 (Table 1) several are related to glycan biosynthesis and metabolism (e.g. other glycan degradation) and lipid metabolism (e.g. sphingolipid metabolism). Sphingolipids are important components of biomembranes, mediating signal transduction and immune activation processes, and they have been shown to decrease in COVID-19 patient sera^32^. The feature 3.2.1.22 alpha-galactosidase (alpha-gal), lower in COVID-19, is linked to several of the decreased pathways in Table 1: glycosphingolipid biosynth. - globo and isoglobo series, sphingolipid metabolism, and glycerolipid metabolism. It has recently been hypothesized that dysbacteriosis observed in COVID-19 patients is linked to the reduction in the microbiota of alpha-gal containing commensal bacteria, or alternatively individuals with higher alpha-gal content in the microbiota may be less susceptible to COVID-19, supported by detected negative correlation between anti-alpha-gal antibody titers and COVID-19 disease severity^33^. Elsewhere, raising anti-alpha-gal titers in the population by immunizing against inactivated harmless bacteria that harbor alpha-gal epitopes has been suggested^34^. Here we additionally identify glycosaminoglycan degradation as decreased in COVID-19 samples (Fig. 3), while a connection between decreased presence of host glycosaminoglycan heparan sulfate modifying bacteria and increased COVID-19 susceptibility has been suggested^35^.

Among the pathways higher in COVID-19 are several related to carbohydrate metabolism, e.g. glycolysis/gluconeogenesis (Table 1). Enhanced microbial capacity for carbohydrate metabolism (glycolysis II from fructose-6-phosphate) has previously been indicated in fecal samples with a signature of high SARS-CoV-2 infectivity, along with decreased abundance of short-chain fatty acid producing bacteria^36^.

## Discussion

It has been reported that the host microbiota composition reflects disease severity and dysfunctional immune responses in COVID-19 patients, and that gut microorganisms are likely involved in the modulation of host inflammatory responses^37^. Increase in certain opportunistic pathogens coinciding with high SARS-CoV-2 infectivity has been reported^36^, along with depletion of bacteria with known immunomodulatory potential in COVID-19^37^. Overall, loss of diversity has been associated with COVID-19 microbiomes, including in the gut and in the upper respiratory tract^19,38^. To further understand microbial functionality in the lower respiratory tract, we investigate differences between COVID-19 and healthy & community-acquired pneumonia (CAP) bronchoalveolar lavage fluid metatranscriptomes.

This comparative study of microbial functions aims to mitigate possible experimental variation and resulting biases within individual samples, by focusing on detecting robust and consistent differences. Our framework includes read filtering, functional annotation with a protein domain database and enzyme hierachy, feature abundance projection to a comparable scale, and finally metabolic pathway scoring to indicate differentiating functional potential in COVID-19 microbiomes compared to healthy and CAP samples. As a result, we identified both enzyme code features and metabolic pathways that differentiate COVID-19 respiratory tract microbiomes. The resulting functional profiles distinguish the COVID-19 samples, similarly to the original taxonomy-based analysis of the community members^11^.

The differentially abundant respiratory microbiome features and associated pathways identified here match findings from previous reports, relating to changes in the microbiome’s functional capcacity, such as decreased lipid metabolism and glycan biosynthesis and metabolism^32^, and increased carbohydrate metabolism^36^. Our findings also relate to other characteristics of the microbiome linked to COVID-19^33–35^. The decreased pathways include sphingolipid metabolism; sphingolipids can mediate immune activation processes and have been previously shown to decrease in COVID-19 patient sera^32^. Additionally, related to the glycosaminoglycan degradation pathway, a link between decreased presence of host glycosaminoglycan heparan sulfate modifying bacteria and increased COVID-19 susceptibility has recently been suggested^35^. Reduction in alpha-galactosidase (alpha-gal), here associated with several pathways decreased in COVID-19, has been connected to microbiome dysbiosis in COVID-19 or alternatively to higher susceptibility to the disease for individuals with lower alpha-gal content in the microbiota^33^. We also detected an increased potential for carbohydrate metabolism, which has previously been associated with increased SARS-CoV-2 presence in fecal microbiomes^36^. The findings from this analysis call for further in-depth research on microbial functions and host-microbiome interactions during SARS-CoV-2 infection, including investigating the potential for probiotics that could be utilized to improve clinical outcomes^39^.

Limitations of the current study include small sample size and sparse clinical data. Hence the results could be influenced by overall variation in the sampled microbiomes, possibly due to subject lifestyles, location, and clinical characteristics including different stages and severity of disease. However, abundant and balanced case-control data is not always available in practice, in particular relating to the current rapidly evolving pandemic. Nevertheless, computational studies can make use of the available precious data to begin unraveling the disease-associated virus-host-microbiome connections. In this study robust differences in the functional potential of lower respiratory tract microbiomes were discovered between COVID-19 and healthy controls, community-acquired pneumonia. Furthermore, examining metatranscriptome sequencing reads with this comparative functional annotation framework could yield additional insights into microbiome alterations also in other diseases.

## Methods

### Sequence data and functional database

The recently published bronchoalveolar lavage fluid (BALF) metatranscriptomic sequencing data^11^ of 8 COVID-19 patients, 20 healthy controls (HC), and 25 cases of community-acquired pneumonia (CAP) were obtained from the National Genomics Data Center (accession PRJCA002202)^40^. Pre-processing included TrimGalore^41^ adapter and quality trimming (-length 50 -trim-n-max_n 10), and poly-A trimming performed with BBduk^42^ (trimpolya=10, minlength=50). The reads were filtered against human (GCF_000001405.39), the PhiX sequencing control (GCF_000819615.1), and the SARS-CoV-2 virus (NC_045512.2) with BBsplit^42^ (ambiguous=random, ambiguous2=split). The paired reads were processed separately, individual reads that did not match the human, PhiX, or SARS-CoV-2 genomes (278k to 50.7M reads per sample) were retained for the microbial community functional annotation (see Supplementary Fig. S1 for the filtering results).

The KEGG Enzyme Nomenclature (EC) reference hierarchy^26^ was used as the functional annotation tree. The EC numbers define a four-level hierarchy. For example, 1.5.1.3. = “Dihydrofolate reductase” is a fourth (leaf) level code linked to top level code 1 = “Oxidoreductases”, via 1.5. = “Acting on the CH-NH group of donors” and 1.5.1 = “With NAD+ or NADP+ as acceptor”. A PRROMenade^24^ database search index was constructed using the KEGG hierarchy and a total of 21.2M bacterial and 53k viral annotated protein domain sequences (of minimum length 5 AA), obtained on June 6, 2020 from the IBM Functional Genomics Platform^25^ (previously known as OMXWare). An earlier release of the bacterial domain data has been discussed previously in conjunction with PRROMenade indexing^24^.

### Functional annotation and analysis

Metatranscriptomic sequencing reads were annotated with PRROMenade by locating the maximal length exact match for each read, and processed as described previously^24^. Minimum match length cutoff of 11 AA (corresponding to 33 nt) was employed. Classified (non-root) read counts (6.8k to 11.5M per sample, see Supplementary Fig. S1–S2) were post-processed to summarize the counts at the leaf level of the functional hierarchy. Leaf nodes contributing $\ge 0.05\%$ of total annotated reads in at least one sample were retained, resulting in 633 leaf nodes to include as the features of the functional profiles. Multidimensional scaling (Matlab function cmdscale, $p = 2$) and permutational multivariate analysis of variance (f_permanova, $iter=100,000$, from the Fathom toolbox^43^ for Matlab) were applied on pairwise Spearman’s distances (Fig. 1B).

Subsequently, the profiles were processed with RoDEO^28^ ($P=10$, $I=100$, $R = 10^7$) for robust comparability. The per-sample parameter $P'$ was determined according to the number of annotated reads as previously described (in Supplementary File 2 by Klaas et al.^44^). A two-sample Kolmogorov-Smirnov test (kstest2 in Matlab) was applied to identify differentially abundant features between COVID-19 samples and CAP, healthy control samples. Features were ordered by p-value and top features selected for average linkage hierarchical clustering using the Euclidean distance (Fig. 2).

### Pathway analysis

The KEGG^26^ metabolic pathway maps were utilized to link functions with pathways, and the pathways were analyzed for changes between COVID-19 and CAP, HC. The pathways were evaluated for average abundance change as follows. Let $a_i$ be the mean RoDEO abundance of EC feature *i* for COVID-19 samples, $b_i$ for CAP samples, and $c_i$ for HC samples. The feature score is defined as $fs_i = ((a_i - b_i) + (a_i - c_i))/2$, positive values indicating higher abundance in COVID-19. Pathway score $ps_j = mean\{fs_{EC_j(1)}, fs_{EC_j(2)},\ldots , fs_{EC_j(k)}\}$ was computed using the set of features, $EC_j$, that map to the pathway *j* (considering only pathways with $k \ge 2$). The pathway score distribution was normalized to have mean zero for visualization.

Median absolute deviation (MAD)^31^, a robust measure of dispersion, was used to identify outliers from the observed pathway score distribution (isoutlier in Matlab with the parameter median). With the default parameters, an outlier is defined as a value that is more than three scaled median absolute deviations away from the median.

## Supplementary information

Supplementary figures.

Supplementary file 1.
