# Supplementary material for: Functional profiling of COVID-19 respiratory tract microbiomes
Source: Sci Rep. 2021 Mar 19;11:6433. doi: 10.1038/s41598-021-85750-0 (PMC7979704; doi:10.1038/s41598-021-85750-0)
Supplement: Supplementary file 2 — Supplementary file 1. [file 41598_2021_85750_MOESM2_ESM.zip › Supplementary_File_S1.html]

Javascript must be enabled to view this page.

magnitude
magnitudeUnassigned

CAP\_10
CAP\_11
CAP\_12
CAP\_13
CAP\_14
CAP\_15
CAP\_16
CAP\_17
CAP\_18
CAP\_19
CAP\_1
CAP\_20
CAP\_21
CAP\_22
CAP\_23
CAP\_24
CAP\_25
CAP\_2
CAP\_3
CAP\_4
CAP\_5
CAP\_6
CAP\_7
CAP\_8
CAP\_9
COVID\_8
COVID\_7
COVID\_6
COVID\_5
COVID\_4
COVID\_3
COVID\_2
COVID\_1
Healthy\_10
Healthy\_11
Healthy\_12
Healthy\_13
Healthy\_14
Healthy\_15
Healthy\_16
Healthy\_17
Healthy\_18
Healthy\_19
Healthy\_1
Healthy\_20
Healthy\_2
Healthy\_3
Healthy\_4
Healthy\_5
Healthy\_6
Healthy\_7
Healthy\_8
Healthy\_9

138139038411462222254135692500913004614263148944942057639690115394828822241634895105963114285667690179691922393022184922321450818047511310036196374115574755850598778980743345862287418613112822379141592030636294177783641081029506171500097109550471182810191298381907618658670904867124884109283606608656242725907011951178672676726353

8781426041232372256795335932034159196457321667348234132140363264371476781144111247052271532828254271145201492801218010579870186062199530202452280336107431466418952996360940042058926726472024151679101693132165119728102091131959692007694512928599620941137868325148897909107741
138139038411462222254135692500913004614263148944942057639690115394828822241634895105963114285667690179691922393022184922321450818047511310036196374115574755850598778980743345862287418613112822379141592030636294177783641081029506171500097109550471182810191298381907618658670904867124884109283606608656242725907011951178672676726353

73993565731411036331978085287855527789792749932473700959401674271216013630675063449322929397321855885110026343635027092131126047206495789244145024891000830832648340138566855429041165676344832977979345045524721461818673251
6792822213217135932942372415381327236249135541039241533317222722354147595114121439153113

67275734336120640285427176444216221627149661851146850212839081795532261732412091824762402114123323212122182723512429

67275734336120640285427176444216221627149661851146850212839081795532261732412091824762402114123323212122182723512429
2742632288313022957713162115542324461211141411

228351621610574281218233977143210233280128228984422349741724104335

1137262124924122141921436104911042424127411116421251118432312121324

2122422

21161910223211174

1413424222122117111121112411

112111311221111111

7

32828152177

171711216212422571839201763161192280754428145384837131111227710462135751310912972

65224712113311

232193239282311446153191441641125611545443485275524553474489242440124111151151139382534586993650976557532513217403443

232193239282311446153191441641125611545443485275524553474489242440124111151151139382534586993650976557532513217403443

1813383651761045264221265133841341025730233113793241171849463126326332026166038393215284201722

386243891714836107727421122584311613947514222050360410212911368524671017522679934342618161010412161720

223725421425614125331315141

330631681459808912493910144422697535241294281611272362535926173126270045727349133001542117048775333014428901437192062017015187921791634130142741441123116201555385815622455218215631589398013921828162117838381261

22438224833322298293814912342412232121122416772432

12336224827312226283814912342211222181223672431

124711221

1211111122131

2361391427461027352221351816664870236370878115611781816107838335183138647373311006888916761118450322816374412546173312490354824461868

1156222211221611152311

2411412144315214224211111

812251113

230138140825101745187264126854365815344982113650671723503376641372111002188296401767101248111001013311713146313610

68107729144111072204317292556711024641704517825561164553471137016910232319332813156515387301039

1710222153831323665111172441114115531610627328532118

3067173910288037222386514172039333259128727654222323164225292977207944797147126771460113048736280813927427901547587694598901112620128137981407118516031516378914342408200215321543388213531771159717348051190
202139256487866366451619563406814843201233304357109234610222356646976529330325362897292210225114170216943760031129634076938524722031827822995362198257

11173885228933332493205133384011162931119561987501399866451406750140271431208215151202737910378335423413833364210532496776692894

105121242471834119510751

52912622317313171101395201053261217624214747123432132352719

2963112345781872721514614312344107533690363613829535817226814281319101719575305154125339625446099453713231629

11111102224

2914963218713113837635176173446814627161473511121054713225013475911155772181104834

59110351152968559381752161652331113171411624021135271132123

4292251941876412531294429382635571542291048331463111321255127611134536

112132216122411111292372223

142238734683561327155322562419322433955310612

92415315296952864031764631252376844117654826663226428311982303371484761951628113533320800323324123711356436103

962822414214161431316297439963388218965632101069441131671046822

127919134107161839113910311211932312195361139313115594111496102121098652242615512015173475812021711039163005231685005322252350176814823858995461907108927267313

121232811321712712121411152327124

1119141

81385101371227511173218392967221131346142624121512128122036606055377444529741436311471

1111

148313313629121511322321048913213

149965245222833961112182

184136111

23923301291707115219314243362082264514141670237201352112

9162722143724586402510773029229826995132464708113062161467101017303413711223217182819125926

1611631114142217113118661111

333116285258145144245447121935131256128517237

34125228878712365918283084432530594613141668723016552718851136521493725836332431486552391937109393530408182533254428

101018107596241231192542361331196498514410128236129263413421613242706785893380395076537023552366

263172611122115481522234

126177777246510836115213711331343271219171922517132365994351015444120715122501279

175701113241878111590244262564479118161671525169371343104617231456917736291849218142631271152417122415188745818

11125111841

43141

23114

103211351327215111113413221222412

13334011712187826137142320113435

131123911650322221024447324978942112934139732173333312471

144313211015374352691517210224411624871335441822451495753

272611251119434791118455441644273501012061024245423581241692982657312511910620110462984541715233431274010231241

1

224213101111111131211

1220881488131641510676123253144176471212311229113320132514868225711201

222631119113814388811426328314173333131534438811736117313337

1412131422415

2023210136219253283821182118114201227021391613243452

1111146715223373113231111111642

2418381764810237187831269442919261138467407112120142771861056614881416302412711331318203224726723

22137311211203411221151513131244332241

136323311111257774336191151181121115125151

136323311111257774336191151181121115125151

111561311211371326216

111561311211371326216

111561311211371326216

1
677635829744178727289672411831344732945766535369269685168826064954363274220019502149532420183460859755998213613753041556032336933421041034255224410241680671431108325

153824177373132433316210437718451425444352348213161112218226

153824177373132433316210437718451425444352348213161112218226

10535225794837107047451232647307643238121146701417494495483423170194385144524202322731852404947216341457554365305014174837

8621732623253261313371119630261195393102404133294693811594302139484358928103821194249414215322389138416

84212716143411111112

111254212022138

3110135618821134424121852113712411110

9131419261054325697320113617866746593931816629521541043025736926191573429112114511110185981052749

21135505544412822324026921268945334336211

63131151951331024216663311534541112262108201152114308391166165106343159787182291625151349473161441123751532112

6313115105391023916613111534541112061107101151841872211661511051231293871741882211104646510134111375152219

9943522111013012117141223481183433861113

49461542583616452923555129115292781863729126362438062051274962923221616054381224629516137351511322654099827221995291704612438590318316530219959252420477270

51382516522184312423235111692062193243130764890100153610633923630237359182651454946097123197165472121171212121021503013248193

1

1114841115223214244767242291446146130111421182441213

984815471521927621498622112339982513142662278611938223767739815153392758116513929326

21111111

4162222723043936919439751220234173176263161604004155743151247631036367714116801239673314410471421144111840302045243698441925

7872284311881362317657329291799113247348421091558481057263711626

1111

11112

11146733210752198148521615476722934147241419531451254333011413132101911510022161823121839393236

33671525323101239274210315920284015243153764222387021533762525586274425972113326123520294216

33671525323101239274210315920284015243153764222387021533762525586274425972113326123520294216

29421022385565943111171211562436143124382311316211412522172

21231314289514512356813734990411762213321021912426129641102316

12851231211102105581411221186925123335

21452146471353410245413691211131151414121373773622

2009257471359175014722919105323114613544114121089961254710841365419022484204421004398519026145205385716026728631263632526424637666370595638511209116711111950157223011407231614581364338317461830235822728421584
113511113111

3106076115222161422332515960303749254652124712061573203047277546379711211552046152234328281648577218674343181553774581436
62875921266

3021810211512919112533722271048222242125174132181029275881545414126161581714822303611282521923125317614

2127511

1521

541253201112313452719197192291366100683108358794611211661261087182234716817241393434313

114322182822812112161141

3605463112444052535036112113710108736184494235221851710438521617

5218194752996321357104396284357216826552442159911510420311115917131893124249084351808976940213114814153796691004097362076451644116503214105469
21311

32690205251201126374224949711285721337510632548616433942710171447314655843522260140755363762314111191553933212513332128

223415234157537243610146121042754111132310364729610131068128681117721746

1217131421411

112211241038824416346811091412381

6931122441085112718361212123515361487319413311993408377096639173161619121018126235621

31212211

1212903114122112420962271121111121

226122381115

91241

11769483842110891268866117811511292811547342291331022485123410142534713226579319169731075122310

61121546901213337112122271413825149337141

182642914044165069513371

10011841583195678812128421417541853010058887981328100711702138321551530761819621646418233285068170672012023523149165786302521781248610829889531490124419511288189412581165238914981388117312097201322
594121322112141

711343180284129931017167713411358982657352681152537215517212917613324023159174157411150

13152112141301662242111515

471124523161

121

28571363027609891633414116313782227824132586139169982014182298113313

3222834212

1882211842903346316196311751811443191216283330462726

490131440025744550462941386331260847610656017441381612686525683556361187166395123012542961130102803125347165584144326126218417201054661646733810929338741512673

26891311510215342411012231911111621411

4138529821116581011

35111317728678148823114471672639771494332518411063821179151792316715912915

19112411111211271111

51744111347884421614491636113645212281211313159816107793810844838512

222211

353151

4822149582419612281418281264323119421221322102

1956131524445171355322411419137215189212072210132142212146234425112

402131942141206544463821523113425135223281254

852103267714597610101126182477201939862917887623473771021531131212719251424101362211231

16151532215414412261141139170178324178384016024128874781548145229415

15314242313716590117411125232762578105497229141259637493745104104191811971661822134116312

112168

661412122567412315654121149147531412662185263

1

239111

2151372213451030511523312211321311353631141

423144135151123756492514302271225113122123144213793563311

111271061819121069230910102436424353358493623532991851323147425918663150277228402421374162538

22628221172415094115272318317221120273441222641318125436227125313

2231322153104612133142845711723165112173265623148262702399953191523863504133133128120

17424757513568322161703252918864492120604876180345351569884613319836116737115576631281221110121102513183621735505214322517

545142293524371235692512510235417231777433108793185

1

32241121018241863611311711303514121112111

204110213216411196672224112441941218241

622381211411254322217212511531312432334377

12185911218286411514171850113517578421619116522516510551220130192017191139894131521132413342122

148104743376665732289385217729713761747355518353120411566652134411770681184161430221196231332309509183449260253457237285437244110355

1
1764374244330188652847739213189612031823012801541052109786956067537286414361103599102557260662557331223118165147921041667635585719553156

222128120132101231119141222163112

22121223221

11417832433725161121134

1112655024292719855227121911112144

715236291121211483211113111222638463418585546

113551313115103153216242392323311111214526314211

2791145459641596911894841002857401733334902143441394722

1181377191110334182314413911462842261412125116581643

3815013421181013891138209346326310039153106512561949273533402157733266242822327273022416048388249455034278944130100

51321086505751254891478134415408157812317292411

641618569176392111637383115451321281443153797271130355611520512339522168101171042

427263231173274443356531033541000647121216701923252150371834242940263025501749
5845182188958382716087053811180951482418581963921279531007012981109803149916721627932038217919467902430061520172667416938279581535211608655389793264462724936441468825940824519618474014384665875154296505104239866351399245310230930282877589969138925213620578585528213073473980280747172001

27918379361835391233618794751661333102113541475416228158447129710815127261634610262484369198239073153104526431021535836635412831851421849523735816920496310

27918379361835391233618794751661333102113541475416228158447129710815127261634610262484369198239073153104526431021535836635412831851421849523735816920496310
1

207768327597122209191222612144115124902226925110412010264391016130172812337225294671428171628321

259176722938026411429175726614712130801075294613752131464431207861252717138350625228334319383816313790624728220332535932612328946821148020934115317691289

1962

7033051693302721167130516414328454681746221012582115708779513115697013785832121446812535194226110892283548788819892420303791872541403776811250411163901810666688674401199076338278599528048866911486

7033051693302721167130516414328454681746221012582115708779513115697013785832121446812535194226110892283548788819892420303791872541403776811250411163901810666688674401199076338278599528048866911486
1

691303268829171615882801581292758432174551981257511559877342211279511248002982034672247319424910887227522728813890820157790472521400176611249611151901010595686874321196976288262598728031866411473

1219511583256148736712711691291913582311962212556266616146142362081287018821516817513

57971521778673034256527518421146413037178609316841718970492786988898848479019022130398714217415461832558831766237615319261581322771531472614733126533715442075340095568823546417481811506356546145365488748229531336815231719216885269625503238234523652219612474994204291443211271038158580

639991522226122132062731669211913176206206119142467956341931221134181128710
57971521778673034256527518421146413037178609316841718970492786988898848479019022130398714217415461832558831766237615319261581322771531472614733126533715442075340095568823546417481811506356546145365488748229531336815231719216885269625503238234523652219612474994204291443211271038158580

172220401924173661169143310234678262271194801140202411281121439103510117294811342925980172546648821354037

5891319322971334083515211516182182126181129171035512175110155926544171521217552861731513116112113017425

13110021276572958815097196261045161781478352115822682910242723825205293518171011727322223441241542789549631312214

21121030539118192082787771526119827226115930126161364152315171011838211718322321141019221461241312

25094030851119382257730372633412258125156254964180833466822523617320598860385793937304665768493714930425041160548576492663

9430712210322873996911104220085451479641263209878935364738486017218189911541614246344037262407739117515551137717773982197320597951

75645687387975241614231310339124684358221790614444547728451283105041720203732020188142141412

186636382131183971871402912484862133531052858632373353153161177941065126222615814533526782229236371205212919382362

196620461329937363823782923281754437918913137188671093080112893611152418939102423233823848344124573822292602715321930

301191016911093335771830419721444225086138651032180206259143682741926110583115201147630273643291162745161551925151191333

3336702701632101462669246147547284152706319164215313346206112522083075319926816131451233159218513231786144415291215

574245217462429085514065035995551253117655918991232196247277787882344828741890283019591229540314169005798236536441472724744122334150934635208852821190161710226285423490717425011138955728144334487898228413335804230302215978268235494248156323368819506973170202498442315270459157683

4213231021689734987229031681051629812913427321041221280302199994313982105929794305364110125391978012617913819417121511810329414727320712889102

926271267371466511982449222327297318732441523751914114532293143444576596271413524306135323120542131367223385412139

4537113920713262217451081406065143446723713350100215971313311741412188415343911346443122303919323313292233776474228

331868851127396312119811473241121616410272192441213910184348327124215483225

1183456648183138135524438225415893164425763164629221101163071030219892922327184214999034122121103418819274118

24296145308126156493568299911818054195525508162146159182308468372802250843323417778683153811189147250369376382181425281253441263381512277111334

513122222134588731012107614920162138465155411623260616465315926391527191814373567681917

3471377398395712657810220260122204501432381804714053131771981985323581148295572551129681681057960851159144691187891911724101462197814412413060234724474424123148

4162093142231503116368853382610684119511107589302542116312725296
3471377398395712657810220260122204501432381804714053131771981985323581148295572551129681681057960851159144691187891911724101462197814412413060234724474424123148

11

78172688361351185324163129510524410660737258196231531023910587945710169942401964141136651123812207311

3810422193376048710610578281937199131441311818744173217201391271316491915217123927563740323325

1112

1622312131

2

11111465321449416171447204163174501172083568323125828823102217072

33023742532452224307112234180511419811873585324146223210

112186172252111653723916255101369285183254112712131162561142856152112115494290121514352223144268327

126107697545913509168241111114011310046145512651323181111541172325101071129518228620

891022911811931632573391718718178487662148698918127426453071328477286329114382685827224694576588851623057444830024816021973852

34706478316727211033384012555012631615782565164754931458134336701245436784646643759782107313481288242912494296996997412743019468208168476061781039115319071865151818184803209011251227248714181624251920438041428
4812219441268158463972740934158132775737191002643297501946445589102235190756547127116873711471769261973016124123

61234967773761104250490973311594522411821727113991381969179213254386207941260516422272694437774236115166941102659255971552132591282089913985

5902185972363763342449895183715022112616699785118187851701204637512513115618011966461825049217301173947402044999151215821633111228

18120310784111372021651564127414517781061247584513221002373737534721464251543335762954

12312213154710437522016325976215

410351514287692434221128557711100269811042121245122161565291138633

6077112678576159925175712382426635627444663572766519636607388979292617330442855423894332595872216958941282512469303231350460354471610461341267642419366629525270439
201

134131527615357111112114

2415871741846931980371268790158197147146396375242283117516228687991883751221132645286115068791971538165135160323220307933131971543472331855230995276

441112363851810127811512642294731381456219124274110154710133582027444342440363834179453145462718124712031

17645130917975181126320441441604122477717280114541001377367112141105928122131691213128442362261638252826182233282035

9851234623964295594632479739281206132141877334309356030504342114119920660716132482233100552694322597534157134128520573385

12611

461416221174127118998892113436122235610011271143318791439182797510651106238105115667131180212

1
181842392056173402223806259145592781633631752320460260210322142252532425071850951810904251614714444296941252641646854122322814232043027424713203093643171752485351854031018666207262

30717142132212397060314108413133865413468641019446741522952138295321652723143010619192313720411433

888222798312611754727215208155611581406851193468906551957213029982675330293377217858968315559258174741024411419397110211917615021112121017171111125106213588398166148

732122556410145212571712020191562111134326145632221126341772162462615200146393114293322835691319

112111121

4043768425183111311141892273388271061612259227168289367206571912107347441874391269166532730913623142240142223161217

107228118161165525218430487329116151548146223141423142

316611194212631918303251562114522377292195453761093267313332691625711082078144423510851586352417342

14188052452082361423353971410153320331725644231788331115541643662102817824161361131916135178119

511312109402210239821811740333754216319873275724343262212640248319155432301721246367362838163242411544832

27910261529621323181579630615952351454197455904213395055891513195450322172925383625311121914143778240249518718613552832345855664326171119806369410856420476606555132442
731227423701862211159731248529542468911243458122351717217101280234213813

13710613103307441261013177915262141262216221834318837142319

125451176552652315322323474666544733332914662232183172212176980865327171382139249417201625311861143639231621

62814181133181590259261243204750411697901221311731254161219103044104221819152625494619228432393618724

26185451143774495196929181201128381789639172261922672586952033277117212045144332549753411928855051651094913291057

2134135106113448781858311996113812374423146167831559775479105519

55602545901032218582825424013511201284842534064755405071337443014229602517569349563551509623553587348524181831053

193712206252713171642551001340119101672561044231012140281471236820563431170333844112109102351127978102605339642979

12751841152210511352134313342068142187104238419253452102174751614

16181659940206182488133626169183152704612433802324138192147211813147517362676439619011311282416487100145101129359642110

475214210837316361603106392610106688432117672282383223401110421249510413201517520196553445556743041192315516115853

106314644514196601796195283154175182538291184454789565966142363339192553926955163641008568752022241413772711497415428373277

104263814591004710681348601511247813372701004141323322651586439112247916511330896685467911921412758621355714326301935

25830059613728604233541210218446572337103652983616343564171481113210149141711271342

143954928522463506577956520188894989520942200918089424644478331311355361014116164954610758145201775493971941330214462860955343123388329022191016157259053032613943348192461083823131460403231598428174366363119623379255353985020859137551119828792189772378867594209733068911392
353950191122107472259773811611110230915814919178127120367478912778281344516351941505034586958614615894154106101786418167811314874496

1314311630933013727241948431792102442

1314311630933013727241948431792102442

1314311630933013727241948431792102442

1597163228314491315983131362121667531320498318347257599263055222934021178140158161030323575828811231414739115616411814561942724693353744234293362986763225271011213137522
11

893951289416104352047650936542250931491239779711231936153361011251481638846615928264101314566496454919949176262511916123224

12313121111121267311111211

523591122742552356466421328445125581635461116412311222341

17207251385158383216272984036856084301635817612811528373371662711320293344365573338116228101911193

19147413121373193225643260269155027731526612411481231201110029920321216163114139310145139726

1111161142

213117431931414154231146137113221012813

1287231557667301022468737202019324313687105226730193614143250612730381527741220615119275335662749392668913318461530

1287231557667301022468737202019324313687105226730193614143250612730381527741220615119275335662749392668913318461530

1
22112131371298107426248105140547623912794011334137273144436621973830128854449122673602163113286529312470820114419734924423534028619322242720323863100699265

1114110741416617151322411137172192824411664237

541029832316233112544641115201013162130493182234334215629940451021667208125161782728091917

401097292919362521302561487522702751757456131661362321273582614611179608643010264121168135144931801141321831141384315353168

127106781261843532078167178314345910314517164636820415157449944370519238420529513043242922152515310710067239907373161777318422473

1321133131161022231131323179235152513

1321133131161022231131323179235152513

1471141312211
2514761751571537325545243975428537611318359725904802722681562852956721534901112765288881181867811595809419511622492403084104376142905333823771249405479679456127480

111211211211

111211211211

17332262294434809631721201553584237481118219036331697163042563625881939634

17332262294434809631721201553584237481118219036331697163042563625881939634

21111

21111

31195630111462161211
23344217312415042750422439270272369821755970587459115232147882905182041669209765281851178866211505739318911142422363014024326032885243693681230402468679448124476

211314264755726913546137487363147691981321822845019641137785317551810733074101810110

3182547621347529483122531136413289371410784784116193461758541598320166470312248428

2004091255377171323362431664258481416364491435711087054714393147217114311143404186916672120218926032937839224947031231954429431018230885438

93137324013310490291611613201853158219041535904123491153437444701242278536613150189237183518414019718243240186311362311253520

431111221412

431111221412

314

314

69186601103778581621084221573671371102148301139135353636754052211012629332717871316763315286582351261051027630966610023164108266930110

2201134123121112

2201134123121112

3344367226302511725242125101715105689158781932245844163073111902166501286931535313521169376130111726285428229271064
2123387111152121411

16211122211111

3193297621325813441431111122249111791222234133

221512131122132211111

12131152311137232211011112322

120615176531271116

585312074138249533481117416091496734421214105125

1532541899150558923410067215415243617610911031099731467561284361065262829

1

589765318857614511339618413438642217767913672643146222134

1

21438134224513137422131401112111126341013230

3121121480141133542114112237223621211211522132119

2221711121

68529132226222

6

132121

3614224381148331318317931265422465914361116311443124492372513110113730670161555230245765404619794072175358517421944

36141243711363311182963292653194257140551052913931244923618738113630670161454328245364374217783972174338317411943

1112221621342361125164121192413421112211

16611748140650180403146486156473324712001371248469479333619111762111114940655158104112224350798971925483223585584238288253311349402909473355315388511393292425424393
3

805899962721367541971073203851572634945746313360163361097531084611042819115827891213325142125531166233174241277375820416221250335361235130208296322

231179912968113412102

80589994269136744197106240485157263494574621336016136129531074611042819115827881210325142125527166233174241277374820416219250335351235130206296322

85684944378443251053894915301621043111899121620018487261496298948613103242349710871215881460537255797072278957134655315015816221712871

765014218215463167371628175227100422211125123321112437336513

239851139361221161721232419457622226613595081712914

780346136838317103387311311611043110891312177139716193289456210324233834248814578460225442715820188745123623414715514721112764

281114469355274138094333009146124380264321339654407127241425267217217219065774216006481551790810741426281565901542301821828871510632691141944835310358395295294106

21253138932361226263242533716217422122
28111446935527413799233292914011638026429633965440412523942526721711721906577421600648155179081074142628156590154230182182887151063225109191483431034439529529459

24281222151

5164

118965282421345953161934503799345157371871942710632636211612271012415147042110821011024117217693615596134562546425113383947292442963912555822

155807929048886228624227241342031182719716265984882383811094356797132157931872496118113838637106476545576053136514728312821923

899586201332891414813316122813446322951222815108118192512613143095441211113111010141475016145419189121712

12868253221445311447

12868253221445311447

21692373126622396544375741172832476241537712164304334085395442528173571107321078181922177476095747826125922263517692781069117844998966987104315722535599288542211123222245328810250224818131851565813810100807868964813297166704724414705275136470

21674372526616396484375041072826476191534710261303934071395422527873311091521048111895176772765724825945630263517692761069117844998866984104261722435582288512177123182242128792250084813131751565313773100757857962513276165154719714700275106452

21674372526616396484375041072826476191534710261303934071395422527873311091521048111895176772765724825945630263517692761069117844998866984104261722435582288512177123182242128792250084813131751565313773100757857962513276165154719714700275106452

186667106530190341423261583727733323182922135411733443218145105375112321155475318

186667106530190341423261583727733323182922135411733443218145105375112321155475318

885223881211318121119156241181440111

885223881211318121119156241181440111

885223881211318121119156241181440111

3714424758614124023123532617993244035176422913357188271621394530132222015181825620201214715349372419

36942475662123816123532617987243335171422913227187271621394511123201915171620620201114515338372419

342513351224111213221121

3354247566212131512182517987243133171382913217187271611384511121201815171318618191014515338352418

1521227675131199211251211

1521227675131199211251211

40783461024255617244254919757931163394255111112426234535311229202232628171624616112089

397564102415551623422471675742916319214491111232623453509226181821520131317512111774

397564102415551623422471675742916319214491111232623453509226181821520131317512111774

1328211113235222112132324111843714315

1328211113235222112132324111843714315

251
20699286881290255823303412041853403858818021197317131836425059149714331479567988755307051003419491683321980344081850018597229041428417955653886636100158910525968798764729728466161731771570463

24301793231863720105448522964022894611331152124151182755979352652111494101214283530236315210515935042995145353516542772843
21

1101

14310142242004517933934262584316432121245258123211421

232314822218033188824801693408893589211216939911422452630264192815817826182893231479715734542914941349496429712642

311231126012110294921212073411512212121122

81290323337814547262436164323810325312202123195738231058044194358151610264647563326511134182712202362510209516936572
20448841844279452919892981841743345238317061197159106935414993132913081242525386124966049052190813828759663339714629173751827383246176416128525798884257015527708084245797745461506689540419

315320214328225620236584537827167354126584915813716184651100364245741209032118104024334356394014373239111413

51008825441166120812061120162196527101838551227432516419205061531267147283015221167811158111032517158

122121211

404814331983430071312141328234092343181189619134120276848992434771093121516157944592291131334

208783478831943728415216101528224251503610531112772213

371062094544299771414171697134345023466183775228101058511106944776251228772021213618182819268427516

1725223711655142197212267714631501281229314123213512510234

213921826216211

646613781022120959714481213531273282846813282112976771341334

11112321411362143122317

236413226777134621127262011934502062612306253272452437471015240101116182510124555035477103191068123111327635

22994374211

87150221161322573758427592112276471232332311

10614523011483750154380106113558782583114966100281735972691823552611375411891469715541229859539182236971313513426

22122123

111118616312848214103121121

2734175431712673248124001012451125731313442285911912725428819756291545

472355083155110011485721516903858533131732252223254612811473629344551086116

11

133685326517858983439974143191324115124915225623844158123117136223638114276196371314218

224

666625871314241142158202228373477255445011172282471351512451611263161113354527142521242625357471520

1

467441084995996111941025204343031410688161061226174257636012617719221515121717142012281317

293512472651001832031033127890150228232211253822463222613069211426363114466334214299912778849545

18342628245622122148793183242864772132622352211643

16328212253262115871132367380322176221326923112151077

1412893112238125105151132111

3233221158111

1501161771334111221913111369334209312121132123

15110311410612221220213113121

91754817612647852852173111040374890204111434102013163107698346626741266582011711611991411

286334253129173593831328046292949130120521079129847611012112461323034820528815972849201971091413515036411426

35489154105216208401549142141412529664718121139422814241640137843769399165511541241445141722233

22

33361188641634303941522473513183722116347163167441513285511691391211891211738426112

2610223636931040816432220410125510838416765446232125212

931413113217586100281613411436011341757115238117323476110143342406132

11111125111111

1102155157776761221636173911614832111134723

12

12425532248411511881034301593215855424255444702824879296632331723152627260104662912451181122790130541088443

1111

81132241213225131471118601498463415861116118229517142693385877161213101413591688629394

160201065542174161795336145333135146181555213611223452271655274717105264813142610382433157142518826

2184174131280017811114351299622391310161210841841593325437664123135132

951441045911131238138165791712633132532314920141429920114275203784743148155035948188

209127211322065221011111786236213118221411123298228629238234119198379215212231204916154845481534921936158121110

142294192312133423412376224911223121171111

142294192312133423412376224911223121171111

21

21

111142261211

111142261211

141519146151621058274125328228142338228381121119239312621199239204183511

141519146151621058274125328228142338228381121119239312621199239204183511

9571416118270234538515285332108983692141149197342398617383682260016192892492088501361088179341801421612291150146186110671802752882924183211313532002521252283334549602104366
511

419564204461129113516489312164224775237213426137022122452108144431251027

4193632261129113516489361642247752721342612682212244187144431251027

212022630121121

1424742731563136881420122039557663539379613243912173911120392391452291931153310381032
716372432103315753493414413135133351184683414341636

1

521112111113132421171

1211

8331121113112573112711282520632223451889204811174584146141612201021726

31

8414732215112

776840322585291181149151639864117137487799509025226611211955249859415634721772701061019212880111536418610569441614391

1436129101874432951297264485202837122810131561511313138312678131021413831282824241152827673724

12282441413125111056149221133241493343665111144577448851597137192726401133918311918335845

5144191541022714025454474028144059426946351044168844626424653663328574150202240582435532822

13421649156451755443313413126521160333081427211136
415371791918001033957164699107353639110561288107121959648625431156444612736

18629112923

414613791065832811124182523093711026123777101734722117517413

670336105305223103664184380097878996412111021012716441311381641362252802670713299949085391333153379477651956189321127174871881422238534127

2211127411942112111751714511122217111171141111

771411321321212639135

563122323132277210112127151

503224311

21

1113846174

13301195611184521211135725569613111311

171237839156198612314833111321314237669202112155711415257157119216101621161457028192316256211019

11612112224491

311312111

7919488127217473727341111544053417577736770362920582618264265861216283375519102141099173524352743162729355812089177421695

487101112621315632620821584175622192286679143780783438323617633341012

1512211142112852534861461324214211673281449

551367625350191553825139261019214324962621661613241132304706881838323158449187789303864981539101766319080867448811627811462811573
51

313010267302062010393171972029431830513228231822423141503131024967210203615143205141348241124414616

1421554122111646118246179711172248411

241364615323121211519119141615771341403112214317855926641386360429515740789589082840131278036484258245314089163358265857

1518725112511111

291272564453539702172615088234610209541041008613353124075893153505931132738768685929631593841139152194574136682874748510312310115247

231121624193915155382758410411281333221822101313357214252105132843013931634933246717661533

231121624193915155382758410411281333221822101313357214252105132843013931634933246717661533

10885422354626144118386170392226238318371031163072437102856109463815331662212175913109422312

1082211321411416532124136316829718705523794321012141558218793519

3622122411591583392642723293356124264201446938421513

15331122246226129411332226412211

11

22214122122224115111711111111

16132113134225922526354281577911633132611107192359

16132113134225922526354281577911633132611107192359

6214310513211445653745992261044413413113131729278726668154092934535235764713720914864752

10110111413332587171493826312913218066100829133202751831961166173

5214251210115624212556293810100034272672600715401335233137541332322

262

988115523635936153278177623148541114961914124213426633946

988115523635936153278177623148541114961914124213426633946

12112
16974937270035441724147436612320772666010041934202130993769168971025711748226123394795118971660499494424823851610421273532579695710195676613561177123386715292498164882978240801433134916241922866987

146542752568194475711332578817941889056513491938137529981583870248552051721213583857489484093396168164042068146452127477474121041059278865858411127081193543524280510247817631007624665
142964719411909111508822451897341152825181748529111854453616567418377121454765210227188232

233121111

162767123221112

45131710515615882117142511243928814377267671582192646142496422943510381027621822345

2085137759921081192295153915861040869279573237155193167364745276211260352251113425315394365241282520327428485373553315279411390411351779417365

36979571951210318712443926122832298446383226144102725181252755822782335158413732344212722383741527264523341729

52111111353125442211213213534651112712

82103763831002114362922141422420821056315172225308319331138302743208106428564094325315198444025587

125451232718261346202122424864215391991192861115616473776412971481151528812082241697

111442133841

619332365168438854765481363120620443013148330119127398140215127517212332606387289037131219222401947451745468708453946330542731260384572717123126

2145126021124442162223531171132614611114

2122353117289482224022283315223813313011665321228121912143720952263169018262663345447166704111868481351036425513197

3331175510972541492047251928793837217423362981413922212585336131

441235328223142338883653161716116921253434719155181723193313

1338068315712501161091015439817421140722754131669998112101312101011267444740281976

16252214610144111112456311648158131221122312

12205021295537355121

12205021295537355121

226660129156596132010032281767736558279171576110583022111951742187113119139108895154820910483461269543621722967834675938955428141017874452762491038402557860900236310
11211380

61556606491278421775346052344431054147651026365635315466127774512091064808835333147173234212406279231103197150677784326411632061015614557

421165011132461431311261282711221111514

1651046991594923856151953555312238356606119911961421161679134987076811261489318653858103705643713435210831517620628036719620438328523619974491249

2124411116145182
62334621732722434910119153223185106041782913148111666104212638107236711115612

411145268234216929443711434271117134311

22233461325485321531257815229134811121442141427332333914311

32032

2111518151213

3534177161188172135758496278829769221452722664929293216678651659282111230938598214726080283142752302201621112711069160685112439669121

3671268127137211693561071253694122372162732951046884591314212

122131232612197

36726815137111393569710533941223721627123510468743841425

10202212352829628346193211322228123106234418621322174282131805822464923233111721265136522664351832272613341321

212118

113111831111111

635954222

41

432215

117321711796353250310931109181542122561211644131

94911116114191974166512119931114259623221781029291076541519392234204516917152011101014

924241318981145165819328454711153712302152061415812656141751813810651716

111702851115713223124

7221321242

7221321242

23443577426138411112211

23443577426138411112211

3
219359114171092712220141304204957582149523262657219215373744456417437529413315

181281613332213712231

2231431419621127231673711121

19451121112

1

21271122261152121711512211

31341238

221450213

21521195436832406154922111111410

11

111733135236111131171437181121364263614

1121572112402121621110145191213516

56213716101251712253

12112221211221113

11111

2111115041438665445732232262912514

1111150143

1116911

1443866445735422629224

12111441911222641938072073694516135551291510764404718523713942186191152514451384011216788351795559194476113295166

4011188861122419282091515989291914102573017318639317349624139201835851577235101444314242507294365

21287196252295542512

121496155130101

79269312151169321991629226675173312311391393020327101678111652511681

1111

1111

111573890328133541444788736381337324033886251759272916543233223875021223719048921107039101683718645916599967522306717325491548106696447178171249294044028627947818392584152614777657417426178610819736631145248398242222941152

3033103443222263431108332515153049358194122467996915283383252052384682208699451431516192174461561482721373057166459622421253

241112

1149425215817379883116112816627163046521723692396732561195597913451291111171227147379910626

944967111796312112533111134401285575113519652086702320563751463218851210211037921514

2133211411

14213213211081111111271111

2113454122211

315121127701234421773781122121121131113

5889663101669316771611347376332965229543823355213912112527487479489

11103453134115362171341122111623111121353622422

11103453134115362171341122111623111121353622422

1115418902821325013993865163492973209225458517574727165400326938712912032178184481060191009737032558815139645223033168230915441006142384771712022926038664844571793252014661462765411740417467313434901096238098011762801096

1115418902821325013993865163492973209225458517574727165400326938712912032178184481060191009737032558815139645223033168230915441006142384771712022926038664844571793252014661462765411740417467313434901096238098011762801096

11211326841148111411311112314312511

11211326841148111411311112314312511

297721064221479311613472933624311181010172223724719526827782124311089
126350755711525067207441153916391101862201566274099848823251985117137202949210810741327429511809358946730487406464546350245175891036935661125721073248

114126131651556128221173944676021135593022752425652199505219213271087592783803051189634014212132433162415208135413528117271947301312

114126131631556127221173943676021135593022752425652199505219203261087592783803049189634014212132433162415208135403527117271947301211

2111121111

10821973324485121171716188132829322045112016361161475938124997265491620117431771515129136981511160945172257

1367261037747812291523116192101631514118364455523263186813475216944063142032

94619071448254310806641362114269105111064128044226349161791129172828482671203423225

651753861056441311017212213273650521051283240316011715523121625118868666881733120142022202431334528672158745620

17104100341316410942923811191621131816981334819517553112759979129121732233814572759

4321371462555213022252784861418133484620202182867731631936332441697124514338

51441173101510504263465215421556283111460712635210114113764521513

24006718378840073193684601992901263212182117133327333938534994031791914170230690142142422495488999922644541733454191774171667925240256708111681795351410244832948759113359122061154269126240968282294474464106233113372752245853647583124784683894160663545123957653235172876874793843115423928902092661160696241054373605
81110621139894866461338819330947195162426209227752024765644234819152352679548248166076453923111841411472709710108136332777533273316053080324945584594290845793011325456283241366411471438617984710

59562028052620851617649234157172243481835474103239725026555274137632202361852401819731322712746031442261175318220347824247867718817225099466296211176646852257768364922

3159151511232811
58360427851820651517249230149632193441827469103169724166385234007332202291752391819651322712545981410261173313216047523947667518797184819396246201128643845256764364917

722852587958438102142334899715914101316167797103612815181626104523154721386121133

26587717954328428165943194112247876905232471220182037788329677323892451226278111330474593563317707443494763467592112490283682

775147815922253242122112

113184179292682242024223298107134233343023659242299723731222141251815101962

134316221668329934820501539362190732617461206103472710178171686148242112171020173014965161243143616

29816353382016166814419218136132130121949133598114754291101455351033133281064111326928814152

4124412121171

411327241156541213319221

810215112111219179421012589396853689292714915141080132842714119071636911615919725123271239

22279982785311924121

83210142510211114010229619541716133219823201310131212110873206919451156195232514312013318651

2325181934190371947260152732157174057453227183326733151616084724362434489781217479131654782210425483385128122302574

4094473386172591435191959112039164524121912422562416121222910811218829414

2822135283742874511159619838164583731101713591735941034521515914142591918183860305127131

12

22194168331122211

1216282144754548578617413307101825322543332224287514837145

1216282144754548578617413307101825322543332224287514837145

1193390504040102353114145903796447663977122967442282407073204591222001122785646710260889528139212284230484554921172788384995158273913561474967462282181449219685325475190486175759191308200421209393240727223210253088211554182501289228213925248708155905345168145574200533
12161161121612323341413110

22121412494912211112

22121412494912211112

2
261231494010143614182030676776393037218663588555473253731123228631412145751651619013202751992713762622183265015361313

18112717210803114190512713041582323358520285141536142971371310859171683151027717711783

59103818813442141158284614208533410336892014734726914267437457933151212741113162234331559

4119131215212123113111121

1844101522251101853375103181412312711931

320511211221022535241123

1167288173991101953104131543782445863766816066752281637034204561221957122763637910197883719583207484201480754889172668354767157673613401304061602265181444219669325267190471175739191280200367209291240697223070253079211523182477289041213896248655155890345131145561200510
13881361622349550311253318976965480270281112126101128107938421131037331832605430254046380941405719212436765317145146257169319888699197

149181570111272975813478103844352010742111325111177242248131011866091101713151122764271740162226

111221811111122331261113132132

25221211

1411431621812231114158112432122831213

27251328526226065258247189359922174922743228261935307618313011711135170135623593735

125867320225103626401528149171312730329195481311666213678258673229810311719121615141439121561565319821

8745612728368433268210891313942185216360945298225743426120183821955212074341708829741839481040233653981283501595274619451369651998230333691149181155219034322801189633174858190005199242208327239371222468251768210503181565286648213102247053154856344019144991199537

1112111112421411122511

11488111584461271131142803891152326175311278265103222271351412432743722526518

47629241012012492142961411191382115231010157763201210122109162665623221412301831217

12222143443358236141127116121412241

226661324122214036172221151734249111926

149138864364325323185756312341493198132130361511044781415021152294552571115875911719131117

102051452218145615919837684103543353171242455215426221450228

4258111223771248223410583269461782051036563954941444229924

22191341415211321151216791213383116241231141039317

10396619261334181627621019111231017115331173606311113504391343212311113394

1857690912546510233966722925407431209127919671142899119930065473672468450432436330951171231511477900105111423453437167259312412385167469494463425307547192257262314

1224211018576555423281321791502689452292593268277461464122536117371663024461589164127136193203141172151125499771302870100254

19423356138124581452223362161227811721310

11

1014116792414247123123617537214316911121171

712411479121111012776212619199017142316932311210

62264

1022335113225251141554222425216413404111144321411223

31624133511632706632125810992617112626232352181411221101711

11165557351798442214518248112141442394051444501277952813915920112923

93325245711033232962411155583785518393292231411361793

285718152

11448276167022143315516143126387112334134116646916817561257172619

1212358496983535111063112232331113112116197389107648813533638

44334401394187825972413226101011316634115210018705648132233113201413718153

3233612111231123311916129105117122

46101467829155413910161871241511223898181085715212646774194924161226131315102018412068691969

69226327111221113212112112

3382

8363223314811626175312999215833202523521154782154138117919421477834812852141347514413936355

11339584077915618591240940144191082617322457111631384018571727141855151014173429613147

22138237011511113811

4282142612241111941428414373112655102351620113103046108612

32181
2589273124046111576242065926914162563212641514193718372844215115721869188930292343787182218088192821432756801103102164516019158085613481195301164114714791402144416271625174812251141194812332187195822138141469

143281734305422121395111909543621122
4421611978446733303603922589541315185341118313310402397463531213016442425244106644134817345523228735395537591152181128186482171100942741271664809275113

2

17842244355983318591068829114161510710720173916724922737894413177239629306835

119513111111111222129313111211

91721036523826679382082541931132736814373217623377731685520461243413151733

1415111127424141175812181045223255716612132632125104215637031435

831132125611331121121121

118652468981161269188362375622211325591761167523611147257326199331842317615810143534

11

111651141363211211125916424381261111123194111133210130219

2416223324596327121012326357304811171716395127375146132791087513276394

18484221312899141

5109712212155894713751720384436381912092614919133411382671987981523113273517223848303182337

8731923332742281785113181059112567538268421106285127545685869351256521071872669295

1834161422287481319363651211256141791543167145761321382139623655463218213

1561122

11289411121112123111111111

1431474123173622631181312611121

14121531101411819761761612311386342352661417204351292258015267515531916279258163472932655

12111211525830735136814859729241321113741423342221

1859185941516344702084585220114014433111914471832149327021820124314401457254318075707628176471651170216563361258420210610118378312931188231067103512581213117714161130156711081024165210931993147420986841334
132512357331630764126217125131231516131461

3944418511791214194111121819481785113914163131522314187658205245131617303118313211

411122546114434122

565524891210216745176273106262175646810913021204435849761929653115459813424161826453305958141258166492424960033591276

223

111

12223152323561284822

37111362221192411

71232212244413913487231

210231816316315161205011111218514211221111

22703801357827130169328332749573913621522377361973598681632452288537152313412

5215231167122653511693717516390152122646131511026135221811

9223726238471121321431617014184331072716572297614

1885817911147744132191034412134

2

1

12216111

10881424196240141845321693894654545608162650281403649550515398149843302604184197780570259724121739507230292116053382319394393428411374533356380372357549413565255505

302516261195143567154638837344166909012527217252432104410150994486219326172122319104348145145338233226325341140

5228512982432777944612711115831122346267011134112810837

38107141111728231241104451814411485414316271113341122191319291110141113191319102510382719

531114311324032856213107121411222221275222621171844411432324

127162486966306675140122310048348587337349741221363613669766633252314246293726583432542810863922724213504584534505394333766414285852401038284473

67364326472022214493215182836973424226321214644234791115911745165366221851836266742129234622321214321181232337

2

17273694573598951453131222558241607557351651194795182431460122136594764821125981441451146961612935732449416373071

3842222224143

31

771827664848311867211622224082523331317199115213291242283146771137121811192020119829613

9024716381103191114816293171941211191336

1343111911152755121211028312516230

11111

3194219511113

28532210

122237111417106266181121559364213551229225

229121245174722261394231185945735127911214110110114154693727621527111152171112161310114

182411114844

2883901110733314105122451416203241819273523503430172544336498177110388154222169813925131017232113284235522

2842256611392511714121321371117231336318321371215105432586567413711781361691216418

416554722058461074104711111810121014311214472362837267363681715624214624175416317514

70321102742424201910611284037573502449279267653014577361593407341245016351436650829571372578110980406920170928257316817442826119456315245192735371315294149140256

70321102742424201910611284037573502449279267653014577361593407341245016351436650829571372578110980406920170928257316817442826119456315245192735371315294149140256
3215342144378736124153859514133413424114164218131019668109810916251176452211535

4932347597346634690667521122591648043761762414966285146291761814134915526494628136969127125110226169102615357113

35241463734270184202595261541391439184112786362933207368514218998636271067722610117721439704311630131923

197725753780771091816512102135512673113511816119299512414865111454981818129158171692311424104

84903751793372863210931908331919641451933066764299254182992185356182716841246361814164204669602532710539351328970134242067

18444114121928331219332452103777243372766681522192366101134411337710224344171037451924620611293348166312649591474421463202913

110141

1971529438217948160349336622860667200720394778217488721973138413115270482401303852887486813522343911706190124615161456123958143443110101365302833345180317111023311760717387412177242815745191923108956116165159901219676938820658612661684102886927987472090128620573891024382178773039262508129427
12730319211584631611434395701195103013871969190032632309831012976212639225303716339277244204405405127343351377352133449231194173527

11214384251578881865131234244132052056346136762970124841032612646158119
30541665612752457361122371383558174513360220351803651734271263854621245826483264528301586580886882798550359241105806121194073904335150112762593189025762954289111883577687053617231418183644224425681667176116382550

1395612471144111235519533118111921112436431

24111461433527129111215241241

74114

2712114513631261009115117411071123812373522456454073172882434141010918141710534676136912

1

2125722240912221149533326167917232312014226710512233

1831413252096147244441643713644121222120232441782725512

11

148164061021105797313241813965122104417628143373471232405131141712374123551248294484233318281247

31617814236520133111485172638226321274

1

183232593583621502186112251029276518825618824257369158324551032581010197884916740366617610843923083924966561957465954855035437438046155713488216

362978164484311131837815651854262303134719306092237221121441652450302804199149161179532401201473021151352411531174

1

82841112912563571716132220181851176898605362525091309362716538141125161991930114354733111311

1497174579336122110287111055984293476711814412891711761131318417612405136133419176

2391915412127619862179482145125629646685142306078194024384350813042104189851451926174434355181124022103373910221215

131112127811122841015

1

6233646228701234100712811716369111109737878056721532172422063943541930628451863115078632743874332598626106613011216587132112791151149488016901358112419331061173952414368051413

18471549599723412783372132110123138263254363242177125046252824112147652530660918073485785257216325716772501727203254997117761498152987700189399576935356842235228603

19

1025566519613708143215101116194315454272118809253565566121211662174192119

455445211738111171233810855157191321916256321221036115113532135

113712133211117131211211

2143451326115178131519721433398231121829611624373023361413423172101331834937566183321331815712162414931267242993529410927924

9561681128412815641428117801415278393861310734342044262326962431052171292498424927

205340793145091518513282794984728936102227525359617159102618903546142971151313102394521571713108518717

19304174037165341347803298228569341987593304566949609216841330167798144512375323199863793222958417006937132615973982383374219710998132529078925114615389472116712288730275678201414219005310563411374913094117728661061933361200017488684415769596972112420154805990781958772721560581126184
19375777351166571354963303428570941988613358586858950217100330657871944606397223927864174222975017009897148015980972385294234310998733529113530074615819493116863544033259680501418119027410616711386513180118757662771940571203867495884665771856986612453154960993271998872741060688126326

242121

16725832391427545420937209294420112811291422274410117618643584439311

51152471321211

1782322735982130181110819941241434104432813553734710053426358322363113422461199127124411957413146161461654303249643645366249

14215167255635194331964814403161003481052841332494743662141131711315811861051524141516271429162216219293171910

395305293823724921392719821729662544310065906528947604606662911776229396093241692324099721035362943416217785111681803491332372

3492103635027842500502128154698169651741021718697496776489033032837625339670728632022646864998546254191237304227184707237215192177217730041128069264

1
3492093635027842500502128154698167551741001718697496776479033032837625339670728632022646864998493254187235304225184707237215192177217730041126969264

208982344217634793215234056221242713657997587045553872227920047147355011468863229582348221523211720126519413869318118715628213223537423039219

12716512116322756914331283418912411014135411177012487125162141673132142141218

58222312213547108144611108519632215841181912721416442872385236

2282392412222043145854816117190323341624125310153124336428

346262416114894221061313136265795214541827347117575177310713881013441442171913116

471911651016705185421317228916176135626101620712374374468210356635617

35313818414224242144211

11

11

21025342211

21025342211

205565141239150411224428573282690243179751062746883311432510286116726962299606558388776440383677967186417015329814590438413078317667514947785677569801694983398094825387136117597599457042780850551290760947366

3941967241342510073919261119472122117175615124661861146181308135157841412181318161318151616171614821626
205565141239150411224428573282690243179751062746883311432510286116726962299606558388776440383677967186417015329814590438413078317667514947785677569801694983398094825387136117597599457042780850551290760947366

126211221121122111211

231152336441634105931614532272422728524155146120182858501411412189379106342111396431972427

1141183613149162482154442916212211236

121

337171185251441121115116313212

1141141121812131125501

682794502121014193312715210134114155104459913189668008222215481071933610151423318141412125911

7585331316733387223159820764242257151703219131346129100369811123183324234209162045392131222

3782232611222

69283363741859261471976496730951061397223502427833342447781110229955594215073107831037510480146821071258913445686676

1111211211

31310246343245731916073940127175492233201876131292771234118391386234179252291510841034101216151358758416

244896110106111026612239114189143121141283

1225731221333225224138181

1622043181143158201977322530368668359843367730253798423171425721830172522527724882448456681018653251192259

39421045103548318712101268554222132666191203612661091343100164767132711111727181877

87846115347081212162051113616672827122531554911211571283298746354

141192170271181619102621923132464

13410951512831552671631049678811298463029369105264916117243116384321831247613833645819912783943111311910494

2691352137131038537718739111821220420852254930321066861962184351420028311877

11121

9931030312212913753130414165976221971214323162328

2191248202217913313321893171173231

21121671211936611712037107323231102211131545152022132629

8683233577182559373033619016333615529921180772013310896554719441487255114348729134932554923111921361445662731377923618412334721869759120604374857018762076975399529084605740678044431170856786601

3

18711713412482172612214494181428621231893212311310216122

7112326521572111915516372480376819510247222214951915534333351191

32611413142116715221521131532

671255742281394317271178673343625150034389901601580987332701532148119618263481184256965312220380376466480296464354336615351410149839191437

281676864294343502034151717370272733152419235101616183659962554312044233320331253527205029262315930

4510328114133381513222613941076351524771397111359236841323112147310

77514211629111571136121122926433962373112851241362834432551

2111

361915141215617737262736223724141598134461503712327243910981025154825542261737415780521409860657379102448837133

361915141215617737262736223724141598134461503712327243910981025154825542261737415780521409860657379102448837133

361915141215617737262736223724141598134461503712327243910981025154825542261737415780521409860657379102448837133

1815778572171018581714615291501584032335829771127372122422793141726113431931977513926794763129417016762167

1815778572171018581714615291501584032335829771127372122422793141726113431931977513926794763129417016762167

1815778572171018581714615291501584032335829771127372122422793141726113431931977513926794763129417016762167

23826670751269111270185851191352042393157721028951194913508714701402483542153276978611261106433314734407556955152736615062806174552686179920356435750442179532138122914429571800621671361622950635545331173297637270205402159340130273982644622392542692363528458
518102071480012353911821604174135339388511534076441855524013913633011079200277573462622724545501867321056338638026665220926535869456155044648378057052649297656233828346432671009138574403

5443315988313914347141171031224357115126331631256031603072473112289

5443315988313914347141171031224357115126331631256031603072473112289

112113427218242052722436311322111231
14919223416426529821622659072388573317118552264158230443477116119111149327671412491084032344776102488958692120388110461911017073687132741854699

461475742858215797753428184133494112284090374995321241411962222539259513121127503038014101835275511123

4153331565411373313110463484811159791371194881522408510256136343421425

6827832458224520111111017222050521041629365115161464442241538625527758116452239522333133250354120308321437

3615497921845105918718462029282134109317247281151151411157692713119332232431018247133011453

112211

1922521812258611014291485141810821263133111213575191116121489101261183135710

88841424714125116330413261315273998147235171314931613393261938886628191092631212301443134263706541

6668156795531508965104414183477448983776011284918798872020980150202941942913195378512665858718751908882040423557453515414548170021609888901200513210196411000811739208801490918691180381605219054106141088620498146681403013149337831405214331
572361251325371843842921712234522132173161731344103010127103119510612314

63265213314760187166251013625504921513914992232290345998826132241018961233412142

447111803254143084081304113636513934180811120881715611051977412445166472746215722112641531110051319454431537310802299836810300879711861127512389167978687102321894013437168961610813839176889318994116827133911270310352311041319613183

134512186110805367428510126610431519497211217331125518145101185149834453611164

124446125293521111351324276526801616042162707026206173211611473183954011318231331248947224621172622483028261551918

13491346644117121111

9141171214286281221011381368212032013182585402875519710362244096219326827316104710

23913235981131022131611565160516123249245273217983741252011121610512199

1212111123

143333341626384207106507453758489112235248146252411231437

31231256172217211143322010291134114

119170312472111131111511101

56311102113221644103221041111111

24315812802446322326101256814126782128233593792932785537143433

1021161291188565627138361302112327117292412142524225110671015461982213261595112108298318108

1231213211378213151726634361275142496255154141592467295

142

1119111

134226902862373196785531667587816448812627435690870119167835366936501857477357382242516549751152018822313088276650359418851015108411291091117713281822102210136853193735101624452095547857

5128632861391116117491835142246623011391921408814422235166675105300795232311536194016614403154147287197188261179468395318185150129279137132243292215147

314117306749484331012628647358328463214013237487922199104811622321117

622191

15175165713725310681019412338310717961613231116342245428110758839771129810877101554

92324120636704854336299197114012428181351322076798101125910132922444211

1861002205137738937352911161939603211224304491294418161077351338127751221233287202830371843147728658921703607706567577587708679637488906742858484706469521
1469042067480235897335479620581085658628131441494333009123209514104272821471742151678119222233419001682287412726030166361770628833294003342417439889159333935666419593961935846103537130405743007576540853473603679536185388

43110273314711141237664013210744816193972311764436121449834989

1376218324155952602812873481151194101322356012911271433782428561321517914211939121746728

3274671210211121254450195549911848505966528611633854601002162576123342424267229107211672212013395111491486364114812159154156626

6631811357222224511615349661213551104122303133383128194214265613411151851001723781734411811

3311112226441112

19514593528149171293138186047647723471318626162852162412626413418148302117213831224519223431321338922

112111111

2119724833162169034111302523206325220317915277331511518203244897113822257628107404125582822816313

12134126361194756411846242114110455910426214264552

2

3544913381243470674284401317656141949252412264435036342025411551091016191481318111392810211341

7313114356806136522449216146136551011718232471115127471112511331997191121134

21415235181081081661649151233229181425231822619518430723227045771055125697861393914827

750153511906091206119720025810016643629259352319362849360836179253052014066221642800257014651478477240100878770737456123445619695781212189020741847127328791650189230041730182443317797242523

112132114111112

586789131154315797724618131790383175986394726361020878227710652039916354325785513814283733363163517235252324

151624346671331410337517623221316212221111772115354213116

2551024925075633776231216194312318141361151101715419311537814421562386101173172161416151310881242

4263133285138912215712162331153614428136545834926315111528691161684011121410928210

118173684730722270565112601354636408713973010528321548473253739201327221116581226412363109210843643385173010438187495213790226591820175211531796385527262727237924282754121513572235215819901815348019691629

111644222101121588412111111

5521112517725122011311342124212212

11112211215388111911111

166211459210806152831351585642855443249173295804388461416721441145662837389

26277172661112375638911331254311832168165313567261318312

14211245319122117169026426622131118

1122

472171374149921164456161611142111117146911513116914423572376532

16111114111331111111421121

1218443

22133148912450515312483321661433321110854179932212224

1941232148219232111211

112588218519101910302388171323172314822545224101854183523238822101392179114027361543233641382810193736

1

1753111736201332728111912274141016102581210241028387196441935191615561181671

525236731841411948112119011221122133312013

12168421113411111112

7817722101181019317726392705745211465882551639639410844211018232431411114181461425108723

34819516161518112581255931171312187911512375432552756194432678739479451158470134751022921637135

2131511111111111221

321131532106122192312732121384432715131128

5253530211132891082513814885910873622843874015991521207262017241416512720

1111

1116163531111231432164241126

17053645324121114227610252122113111

121122321

12725128111

111511022924112311111111112

822131038247104257911505962913212282601681147909012128656331112307156313312387149246248148507548172271003816553

3302467641379098303610354657131062649137269121049517145122199945237132159981215

191231124106381913412124113291632148624114128

11141111913616

278124010012151001452552182927937441483326647241715842225572551143225502334603425666121541613115492201423134191548332719

1211611765129185212183484

151172152271512526712237242522585924543233201

851212231582531

161472621441261969166443161031226154766030810973116185342532735530914813915631643229217411821622750191625020

433333195558712662889142317292275218944301913524675262512694106141335210210

1847122397431155154786111624321382433426224332428222422

1114313141159144169617651294725635107120929921805346144426873410128219289545223715468

312806434702539399289312642349050962479800224236855973741116512252553072634953726207294082895105320208013312693701961431850835750790576465397239479432519401622400
155221114013558122391

432150153343751116461810324033448111411113715341354513943

770102137513921230397231713833621199267191637125

262773684532496378280312519348445960679753206189555469739916511912432972631403326207293802895104920077823312683661880428833835615777545454373237447420435401617394

2851620230455289586323703719274332540120638243576374612155471217185025926222758269187099771282824104010911951461027224296326348595414249429296272734293348349492186292
34613123561711212711042221312241

331415716691889348111431014731242811139442273818257122239

16

3545191337211617585712121677118624502201828487136875561434373931232164220856214216131

31389993194941212261914823642031791812839182461895672331954711206314211323864412924161062534212

2489210510146484605884567912746415450164894382436312405512138810988345852314113722561062937242351201651638413

252273222644811254128411506636332233439121125336357554261321245271215361

211

74081051441417569173101039103035191553213915112110269141965100131095653228362513215341318

129119057506011719223100423516315711421291391204069041867041485741181351195318447226589102329122781131512161731422281491544051761966624066166

846236421321162102156322153143221431422

9226415754185146544851940731436522408601916121214014389182351114902913253624241031212849202216201320

6891345837251132126520241411561776956382046242625175321105171211013428242932447120615629165383931351140

62

172114151312131
756317968358863666536222833999035991691359127253413994534655866747127037750250250523364341141021007136481165513015289169311421766260831473078162738912267261344782503260168925628823475

62131250063477222121132611133112152413

12

771440331043293952116670171020774122821350214474155284924

452116228121721

36236947412656232418414813939052931201178105051473590175423361335222229182234373133417837423341565301190451615199501625242027692837151035492057237839872259232356422737153308

2832325815424121624185121102526256594166241426655

52394714382320124612326635103382141312111117761249956031115161467136118366249149210

3412216145849271767799528755394810884661711666632016143274279411166113245142515425342360258612488587591537715284371

3211015511512871113311451142101135133156661111212

296264261161519210140421731153209877363244158962317716782113805922016433227726531665125398106848176117521889812234514313864868362

1515106724532331623501691731394241552333112552715719222191122426512121113920172513243420332016264516162112

11
427364451892331012626231371292613289711246162520577731143030746511154153982841825280383932654581311934351160323134443350

23333372813294633437931812481420151496177165341413151916220111215131619610878892042677

410351034313217141325371619373322116471349524872371422254465352314142320322020232724177410710

19023643417610074778664229148311026347871361821411039012927934244133299305163121748642681933

77111222165111212111823121

77111221165111212111822111

111211115121116211

11111115121116211

2

77116572111

77165

172111

11

11

11

1

1

1

11283273549155477573617524391796786891113899723335476710276961146134041517039369742591769148416613264523903292798485616247154096914104076565825852891102371578
3062142132595752844427169472088282454240151631145032023768705472765265419263234138160002191034839129385498210271731124828670243232994085270811402807193349144681183481466815352461461357314482283321801730746218621992223204137811385624403167271772613692327261509953837

8616948275415273442351845392857803972013361722299810140105029531650231300588404134263247540333813129211412382910719392413116

826693475022724414117873828377939217128596722998101398502952165020129932710413426210349424251082161109221910518162392414

826693475022724414117873828377939217128596722998101398502952165020129932710413426210349424251082161109221910518162392414

3517841310945812153525121312613011444691323764316102123272

3517841310945812153525121312613011444691323764316102123272

515321411411112142112
875168811651905203187217517871234947434206466263213171424913642587348313737224295377659348872208216319648254262384193717110291523903423943594399859662672926718133032511662166351

180388191747315223714467116367028942617451882251453717076484760118141011164166269111402315966319511826111082576433949140171371813884558
12

1603447251113591158121337122436850152147131141051619813292791313115005168811328161

4432125081273193639217121431115811

222123783162321256425445131631126431113297

21814003122111043333186143793291324581244427646342547462

91338314651882026551546299546294019824507117122218571751901267931551136165359261632017303028234331713

91357210132131670652838141513171574162710261114135436212107238911912413161046861112442

243017251441269297766472622104147711651216438810310424317832201249277111172718028203127495914485247123523190591748
1

2215122

141231173317115372813352511124262433152371011614610713

5612626528156755567104417142358362102560137331164296387713481211709742727

36141131450212129112612111965111

2452411772171124933411325268165511617131611525

522371127618211411542472434412122271335122213217555657251

3611496452118572115211622157225117421523238

45824321110385429122517883741575111250631568274710311411821741968

33623214169664311955458941311131161072534101214919135425

2513211333510833153183121014117212275450193113241180718792107216131442321112175273134874

11242314312274132411311124232

24131613233683112218262814113187347481531022411418731072114314421810216425284442

5523221806431112

4942217478391505029271134022416783656516228426179236231668871952042705293172941253222557027161788689239992301172242217881211562945071638015257185
1

94361231646174190010711852561128100197191314192614134323722716551104932010172226378103172812151239

123818521110521214521245531747471512711435

733151411320054234718543481517622134025446864610434265223413310

42528693213613142343627133327272282531323

89613913841415373612156555730183022241412188524539716931224142639351265293749302719331036

132116251111215731231311

371181132715312522792331811531233121

211114173341999443142255111149821628351228558471146312

28994063955953913191449294892328351256561081877912217222261089361781071313834

71261111121111

26247248131612182

114

41116021212259591661291143

610411143826525251122461181924496616319448157235256722323682556346

43261352483781581345212497171015991226231100491715612222420715325916424

4225848410221174612019477835634410

4011114134663112459117

8312326614368131421541331235737331223492511856478414012410148269664151551530253603212141010

27793385219156811715186127245611181128438335410212661015102191188510816633

211111011081747224418218271021436031434621215424123421

11821332141812112552344134122362272722482325621

1
1471048105234141235211833366431074279209567931217961204015815419266242121691992611101201421105723512652553626217268503983785859544054

4322031547685102240315393614261191

1293747129131166134354141206107234742114450757187141268425622112541931

12094132

212211257124888198392884110942503333915365210637331027

497222262412117752727351557662211124628144252151223

12212521380241124383112518141123305316232913514212341

133129112221

23307897254222312144318695087196121982212112111472

24122684150814616139530811316114228173358795129122

366238627134986017145513357135914231919246165035226112013749175871214872728752122411

42107411137549098581075634321299822212431484121913233322527111010578634131666615

122251124181129611111141

12120121

4155331613111411552171034142161511112

111112111221
28558882536803374781754450398227887211132331251918293719424911127371333052537729532045792643583254809265341065967699501886674401117111471100008344112686560716817992927911675114971127011188723765051140283999158665018415845844748

121211551111115323255265383234
6027664100413479513354241907151882816901848199215964239300021047967239055296854764855881052515724382128841279760101599117206066161615482708274335333447156033992176223741162240231510946318161839344

779811516927252303011506610561880812251033397181431865834171109376151632541911463881841523130810941875171691616631057113518451129112048852048151910

25161251341621311111411

221111

1412121217932181071661804327179211622644220112884115731441102201935561741261133354125211395112421696828525135352757054124966843643276839643311652717336754

1114215102228102254115126106232565161523714123212

691871732061334257642936089010610617922275933611341891668615687171724712051586025714909003623121160492190338292384315539109494224273435404353156196398311

436768310183411411482817154332060110113113135101145652621254318313817111341261477

34118101124493850645222114314238197132174713224123317618843792596

31213370611431645110419785939974234345173722141283711633631575011578478170707783856160495567140775479

5931523675512612461427845231310503717103444291591414209133151910107834313

33244286121123012122103335584132811826501637626611187276674516

1324893140118921013154637620233613107131183523814892023112018721530323133182415501433

13214162141161114141121329111212121

33161721412817210871217264534827261911014665444910530619338653136819673778814632156183749324932225421

665111615123361522317192316312511241484821447432161112

2222115941291743987332411155318553714362316110

2214209823212194169253472091630343388312981647336123417826575153771637234896208367212465240151353711202834

6215241154321922521174518761334172464631428559111520536859303814272845194945814439611076288736156831240

15116320827133986522742210240215132467922181472611151439704025586148415779536290395811411955

124111121116

2211911156312

5275214221895589271659511941011148281113102091591886132961426

111

11115011243411451144113221213212217

31115411682121711112344162218411212152

4213933132131113412315772113411

121311101114115311632539523135872641201968582316262211360128111201312713273168372050483343172615522436

11361322243521131

411211514811

11231153111112411212111

167314111112211
2795687487270333333380311535588815693495211829164455202233158498103303149693345973673661390270716065946960716127636737927419514569009662352024943562015282653681418049971077885059426872866157684355561209768395404

62

19441241312515310642444341471236111035726132447244

277588747427003331337631063554880563241121181416445518623268847910315311869214577367236133726911601594195991580636737726915864569001661851594911561315244646381107966970476815025422272096089678755431207867375299

3824922221828

12611111112

493125312216421151312196610932242464232573363277751013141734351117100101

2696981095014547930481307599091080424234918989254644472311852185716759010250200245092218072934488423410899306145746111416828194418131535978884812761166217003506168923364025219110442358740062998143518073109222520102573416325102629
412

84632813722807903199827809292412072917416623079763497211143665134414561197120158312788471313626847311071043182741393239453013251411174812131419349517482004195512312210105413921971172316202260297818182229
21515211

130039211

68642812014778661985909916238192350431577295174148241023346111841446110511300818776645620932594536989219249517150142713021403140011801297347316811988192612062134102113791951167416122231294817872031

1144185848914053781551445426115582501321122222172181151515

154341914918044532714365211832111155332653817538161411543110265135073202112247

5612213102046352113552772223363142837711106136104084735662996701855872232426411181153443181153136186

261235813642267229578107771291471832779815884413933042216885166476658565830532098360919487592130145930094777415757151235391522048645412229337289175847691753044384381632277578838141511385023903131185692400
21674269228175133323365511831151152031519186171301310272013276103123

14011137211111

223294921360205151126219871617614333502043104592213321678846371713112101413117494203128177143155215

3417127934534513922311912312123356313323214338812

43239191123548822157878434984774971747125194393585157333921278553616

213121372272

631136125252115612347141091074246259132118220108202633673062368772588611501592122091272361511692791621845230777233

1102311696346101131814458227311265334413138

35636362970145131101926181943297239264223242055225691825116910478158564716

2527045604019127862193665949111215235381189713932885814256766920086609484223911587472131420102615547172121601342261222201421516023

421191214885351118525155344352115316971072148741113463582145

1271192028622167168783328742337347313021934506375113417529036349566503113940514472224525292929562438313325423

8026224013428187252121871181259328482050870441574331568158674165444228600175118151340701316738743714349785623120358232052521932794911722475349243248813732416819712

11321123115657322144251368411184913256633647439

9211022131501113714283114591419731111421331154365491116

35651666148024194311539663523152107119294674543452447695292162603126351594011222314215613732482237843837291733933136372138

1237411227282111385328122018142111112252412

2636374389228181027616533435131850534411829342822562315712028222319511822204126181813312016

239233336113

642261166364743244449833363823113162221441221043691510372421

22712026440872129627218264642

314123153581511823972131242113711670232267351343613524762

314123153581511823972131242113711670232267351343613524762

3141231534813114239721312411371270221706351322613524712

12421041561215

3041101645057324011441404274419324323122861754820526219162137122731271561032162239131261256217264740284259302333352471217505355353682320385154432161409

3041101645057324011441404274419324323122861754820526219162137122731271561032162239131261256217264740284259302333352471217505355353682320385154432161409
1

71312832215101131358263558913354453123542132726541603549

871113481248728121127389351623621910146215845810161168131112834223151398413410

13214335455131434251434132154915111121572715913412691278268

41221

247991583796922096373911256166228217212532509161216130459510247106138811161130125815046216255586264240263302333417209463329327501302368152409157382

5541762154437133282161118466793725140482342408712936044773191953061891903813851214521023812586151236220549114835024405047948108703809296816471239601707603618710849
111

511312221286232111121112311221
736912021591106603337627439532453266394810121981211048912676701571741124621451286237901652461242405332399669660656286744508538853490548519516663678

16452936457741100417146199139141742281440252232127741422912367934631321251591832361631182151341822431591855219259214

2325196412243212179146647171649102126115334534662737931133632032103521682312224338373592521222671554611836972085117553

29404914142342014154273291591812559346941604215332729433311339229567130534234170204393370368142457319309492295264259271428411

11211123112112115

11211123112112115

2501423272083814990856551611176925631302673711821959616810110421918012020330855124723529232218779485991201977415415587344100131678332157

2501423272083814990856551611176925631302673711821959616810110421918012020330855124723529232218779485991201977415415587344100131678332157

23026971242710203239114139133517419101012135301125445283711132120132017291620311622411127919149

23026971242710203239114139133517419101012135301125445283711132120132017291620311622411127919149

5198

5198

21161810745728811396771435121338203512122219967035587619943630134452517481717202118441357537815
422579753132643847923525207863533453196834423911198932896013680361467182552247717843186514451468042985459888346794610347159821175202333771734937873360377044296442414623605807337938036608367945543126608626854254

2980177027654859389111325624791834162391720210515661786315441101198995106181760437059193366906374715213397415656134475939231366362720891928200722633884206310503062187419723236190321962122199213462037
117151319112217244811121121258

1669215445711276635797144414212285548838924542823252315128103417144

343671343954741212930388313738811

112

111311111337776522237322712101611233231319222422244412124

232273733139316823252271020611285142524788417556

23317211162201158394669595826595111217113391114377142152384535098193816432491155505415

119314072420467035719634791791355720315071905742162829183745786551593624463947871232147872725026733253929131181935757812743069170014511469180125671772865269516711739275517771837998176810041851

177462131004313546619871076761817813381337523123132425

4109172223581645810120291143921172

7544123146848145179412389126903408121521613833610943324116371341019

131126453711101528215113211422144

20522126221

26324107

28287222497527035416258188492513454101215890116319110217222017854432831862156091188773554345425733564822132

82511111173213241114620183111421

424104412618822124641131461

2515170123885711912111212347239415186551171514

1

11131653180541049141181418413122074112479175453577010389124171461918103187810

7135856861411311370721112171958204621147242840373471285234968468017125318243013494917222858167920

991412223102296221662106015509162511313894184123338601010203771688151418109551138

3192121742214412112

23601015779237379864510337301384317727151322712141629131311414974361111823822371521103721208810119922652

4131104210451012265487229923231312572

4131104210451012265487229923231312572

5702821731244447253133555068163404469270251122711535182812069744061122153880190136834594120866129233329655210641268433359669839285165432361327737307474329345352223

21936

5322601471209437178110483343903404341051271084961192242141938318871593754133416314928970412626826751810460212257319484775164113320292260593243363281311315152

144161912111112211221

313613216206162176171

21613

11483315163231112644121132242441113

12614451811382185224211501156111234211131014385102484513174915101271223375164830275929678153139

24422453792134101013474139653311035929128833215681129387938211

1

125428351614538973493108473836121972511115129372761201545114434568472335224428333618242010318

2362124210174161020514712110525235756140536682311682232178766129416111216561340948241664910370355913817

522161181151257113511334213643328871621039118228375386732422374319739117173

184198209152551447778109102714483415452220139346127122458637675121821666324386604

24815272121313424214

21123232119

73511355016832443742641123721516113138793717201212315124527920367297201245145

1211354892311337261101713811038762172001122148244274163069929121133

633126613143113858313171113154635113512

1421541181613113123318715663225252823223362446651215511119961168146613
6245897154166229190613348328749461198121214410511588120643155745414329621257100725823453106215926331418103694678136329461353736136113161661168285919731097139424161289177248736948841965

11769162217341521013431275451172218214

4312

1

745571873238352522721694361142111746139523775225451221920635132145027545235112380169801403002193461324641822283982352145417668336

152131313121137121171136232443113146211623132120271

222345612166074273575137543371219825741125221132

2127624463694413227142415221352

1660153121111114227112321184

211

21231146851132194142121211113425122912

128814132340

512313601215618041267571828231021108311091681867141285601402019011059512012264303429724842010639123913151162638120090314221298708148088211351878978151841034947691589

181213185525133190314911423313383233632

8213211509191103344113321421241213311

8213211509191103344113321421241213311

315091110134413317211212111

8212118212221412

21

9125213186212117291321022011042611749811395175136104712
1142430550449012767605852894110853398234867483468213085111885546281715788621459411199893654329071601774094702642636324224868134742110225135984065390911491311738875592472261955398104446749401540306358476456743672669553104526

53061121037711405582236923621593325311240376150757906392197166854442524443144561826422531067251234461038138516742811931243482335733584927927383047493440004959459646315773349834624951414848262934588550483984

2166981251555117202101613316326847221901831967229312531211512167171219271829659198118421252347

4331246139130161662892454121343226105712117911132237371249322622

11273221412129221451984

962115741231263222731221218109932752651311724321

211123161295133211061142232439101512421141151

1721618582112176211381311113151124133

661122131651111

6636511

11221111

11999242324522162751011724522011528144681062593783354713617150601411242111832151151601436851241381331771631532221231301581191838518598123
5285103913765130758213666360058832029513375950647746259195466534347519742904369824322501044250634381017768216741611878231382235683569911227213035491539724941456645605713347834544832414348052932583350453936

45661105329618131281283471417124643111135111113122523359291564

111

352610101214614138569631387623774446252220651920643712386319402613251814119610269818634234137856

34724176293242529227130943261525352778130469415274562831115193441675687741155518154140638517549455373328014111217814183110

1724122413031623232217183113612

96713841730653316710071329157124911811228109917413935059471373215215287191709780339381998324231184106360107118993113626328561256104012401064128214507958391057106811718041591873890

26562211478129821790654851910214141153943651723272162710866350553416136332386286021152679266116465639105119

16111121615120129311529272041126346177110276351415

121214231486325128152145132111110222

59302125243921392112751823141254042451453411121211346134812526523526101416418524213576

1122111411

29757621110745715741561190022614842160213037235114337116323832250251220053176525011615289171739658204422911168621520222337552118581834338825443073317728893701236422252970264631951687380920302705

212112224652216135162223212212154314230111421

142261321564311624417212132135021171221

68761383160815317940541732771713314718136914343794223252581655

43116825364911881016476614954616236672815932361253174192371927246344337121013131151169197763321067

27182942431505194599417743222

2912112222352371311134411111211

813112734811

813112734811

1132311112111421181121325410312123
706410293245611650615836506119882642111452503716484593405681612849164252235596713510341844913322096362009612781401128194578211365343309791612234255436295411144272127229

11526556938811124810277122148121138102219142826325614154323337251361213621930611812

31122243811511221

115262569387111247102751021461211341022161420263156141543233357513612036272861812

14563337945126118422171214335112571912224961914931013311119193510226211514712123349

14563337945126118422171214335112571912224961914931013311119193510226211514712123349

4667625223077832883173424158135638116715744023917844192225998156166324310988014339105309334804359180653749146111720423118268427510810617517122299758693
2421473224311097913410933110322255740365862529443826442103

217141114253191

1751141114192112335121221811422151

428

38042396887210556102622385964541841063471361412826626865606716668465373567111727291121492126379767514412861922474691405111550483157

11226942612413192111

1011152216871912221189710352421515647546137401217331022831128534141314

513361495301224240165114421604722231118596292715619621740645928334113919904938251018913751612114

5010911341092210143918699814155175821040150301691116102698922928722526154692821139131594193092414

1441124115173212321221111831

110633073321315513158531010114155721841931911171105753851676536804122672013370109227685596508596721209882983051051062211051744018437112

5226332960116172102335219132216129791963311282111

221832602063711513514116214711227123171123191542422217

611473123391118120614313215431720953761393111113224241101

6113557271291071564819293148252458172467364234110883525181759248133358863222215

141

662710222353352921856911920561311129658512016219234129424667321191155253257655159908461592158677867380311363686

38126175452842217295922238549422026153342233138110106147447

484243312714815132810219112515222118133513185132275

1

1

134461444221221218221132412

134461444221221218221132412

2222

1144614422212212182112412

1

1
5392180834188885117102631921620810318788151521354535527008128275951028114962253804772716704923267795322971006657154111789111999321722514223458414675019349265271946285391593502129277

3451748591454486037131722844366122622832261632351291464116791334831121158138311223340495033644270134266381224646181441448
221

31221

6562161348627497215112122185211333431414510966464591645836183310852688241149222

12221604112129576713114257551113153244332

2211237441316705121

2110211172512331025222

1551238558611156429431938921152411028

73172937411211206103171938818571189155711865596204176114712150171614212125101019622151017215821

413125326181613936310212558202334658311211632464514551627579162916208682411

42211234142

222513310242642741413110311146111117824141111

1
53511802934387891088812732081466605613148720914163525687505443678631149322484738522062632421342434128299507714021175795675801182003872787973945006305197231807239345412455115224

527317994312837322650681045144394261441207238325247560014023160732728245832061255893146103203312669879370305117504928395711023042004792944970176951143947715229138269122

4629224078218719610368123618138143086412236210224382190203174852671386114714445542301222992625930079251228510517991178115563794

233212

326994449127696683232928423413146159112518265224961186211918211171712234711561798

22714152217221113112

22714152217221113112

7319223432277351631421031121121733

7319223432277351631421031121121733

1184761631375101252111410941625316163033911112002301010494918103351261092312121828112519264227534

1184761631375101252111410941625316163033911112002301010494918103351261092312121828112519264227534

77181521044379103410752297358611310871013410681194101677103341453

1

4100611336124424654041630114643519991524061033111241122681131892381331

2911111223353121

11221
